# A generative diffusion framework for physically consistent 3D turbulence

**DOI:** 10.1038/s41467-026-78265-7

**Published:** 2026-10-06

**Authors:** Tianyi Li, Michele Buzzicotti, Fabio Bonaccorso, Luca Biferale

**Affiliations:** 1 Department of Physics and INFN, University of Rome “Tor Vergata”, Rome, Italy; 2 Present Address: Department of Mechanical Engineering, The Hong Kong Polytechnic University, Hong Kong SAR, China

**Keywords:** Fluid dynamics, Statistical physics

## Abstract

Synthesizing fully developed three-dimensional turbulence remains a long-standing problem in fluid mechanics and an open challenge for generative modeling. This difficulty arises from extreme dimensionality, multiscale fluctuations, strong intermittency, and the need to satisfy exact physical constraints, including incompressibility and prescribed mass and momentum fluxes under given boundary conditions. We propose a physics-constrained diffusion model in which some a priori constraints can be incorporated directly into the generative dynamics. Using rotating turbulence as a paradigmatic system with key geophysical applications, we assess the proposed framework. Here, we show that it enables stable and statistically faithful single-time synthesis of inertial-range three-dimensional turbulent velocity fields, accurately reproducing anisotropic energy spectra, intermittent statistics, and physical constraints, whereas standard denoising diffusion probabilistic models exhibit multiscale statistical deviations, violations of physical consistency, and substantially slower training convergence, thereby pointing to broader implications for generative modeling of high-dimensional, physically constrained complex systems.

## Abstract (web summary)

Three-dimensional turbulence contains complex multiscale fluctuations that challenge even state-of-the-art generative models. Here, the authors develop a generative diffusion framework that produces physically consistent 3D turbulence with accurate multiscale statistics.

## Introduction

The synthesis of turbulent flows has a long tradition in fluid mechanics^1–7^, motivated by the need for controlled realizations of complex velocity fields both to advance fundamental understanding and to support modeling applications. Synthetic turbulence has been used as a surrogate for quality control^8^, to improve statistical robustness through data augmentation, and as a practical tool in numerical simulations, including the generation of turbulent initial and inflow conditions^9,10^. Moreover, and more importantly, stochastic surrogates can be used for data assimilation, generating missing data conditioned on observations while respecting the sample distributions^11,12^. Related reduced-order, stochastic, and phenomenological modeling approaches have also been developed to describe selected statistical features and, in some cases, temporal properties of turbulent systems^13–16^. These developments raise the broader question of whether the apparent success of sophisticated models reflects the learning of genuine physical mechanisms or the reproduction of statistical properties that may already emerge from simplified stochastic descriptions. Despite decades of progress, generating realistic 3D turbulent velocity fields without directly solving the governing equations remains a challenging task due to the enormous number of active degrees of freedom distributed across a wide range of scales, strong non-Gaussian intermittency, and strict physical constraints such as incompressibility. A typical instantaneous velocity field is characterized by nontrivial correlations across scales and rough, non-differentiable increments with fractional local Hölder exponent^17^

Recent advances in data-driven generative machine learning, and diffusion models in particular^18–20^, offer a seemingly promising alternative for turbulence synthesis. Diffusion models have demonstrated remarkable success in learning high-dimensional probability distributions, most notably in image, audio, and video generation, where they are able to produce samples with high perceptual fidelity and statistical realism. Motivated by this success, diffusion-based generative models have recently begun to be explored in a variety of scientific applications, including the modeling of nonlinear dynamical systems and probabilistic weather forecasting^21,22^. From a physical standpoint, these developments suggest that such models may, at least in principle, be capable of capturing the complex multiscale correlations encoded in instantaneous turbulent velocity fields, whose single-time configurations already reflect strong spatial coupling across a wide range of scales and directions.

On the other hand, a typical instantaneous 3D configuration of a turbulent flow requires the capacity to generate velocity fields ranging from moderate resolutions with *O*(100^3^) grid points up to extreme direct numerical simulations at high Reynolds numbers. For instance, the current world record for homogeneous and isotropic turbulence (HIT) reaches 32768^3^ grid points^23,24^. Generation must be both kinematically and statistically faithful, reproducing physically relevant quantities that span many orders of magnitude across scales due to the power-law scaling of turbulent energy spectra. The combination of the huge size of the target data and their extreme statistical complexity makes the synthesis of 3D turbulent fields an extreme challenge for generative models. As a result, the application of diffusion models and other data-driven tools to the generation of synthetic turbulent data has so far remained largely limited to relatively simple configurations, such as 2D smooth Navier-Stokes flows with steep energy spectra^25^, or to reduced observables extracted from 3D turbulence, including single-particle 1D Lagrangian signals^26^ and partial 2D cuts of the velocity field^27^. A few exploratory attempts have moved toward fully 3D field synthesis or spatio-temporal turbulence generation^28–31^. The latter setting requires preserving temporal correlations and dynamically consistent evolution beyond instantaneous field statistics. Here, we focus on instantaneous 3D turbulent velocity fields, whose statistically faithful and physically consistent synthesis remains a challenging open problem.

**Fig. 1:**
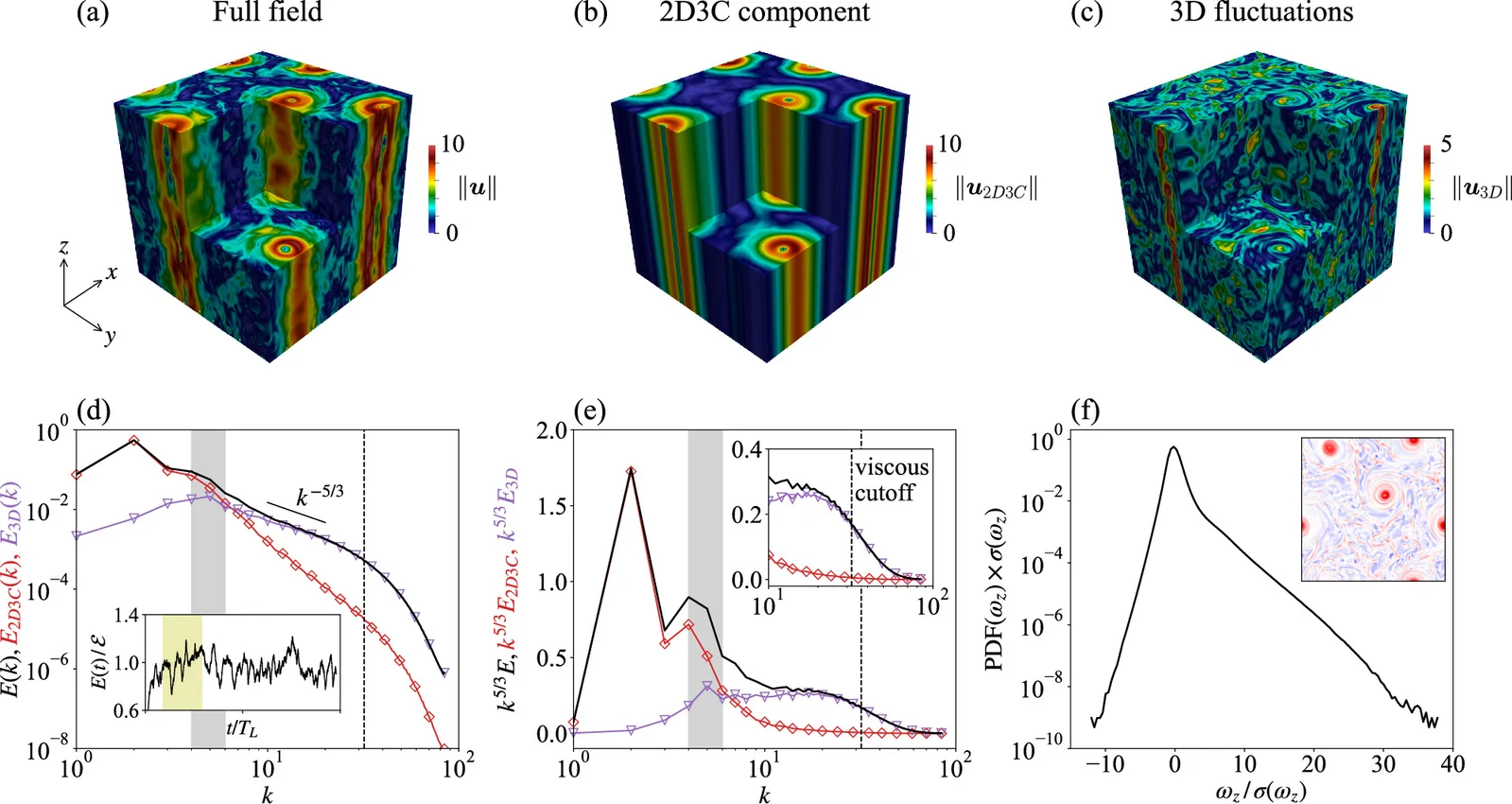
Multiscale statistical structure of rotating turbulence. **a** Instantaneous three-dimensional rendering of the velocity magnitude ∥***u***∥, showing vertically aligned vortical structures induced by strong rotation. **b** Two-dimensional three-component (2D3C) component of the velocity field obtained from the decomposition of the full field in panel (**a**), dominated by coherent columnar structures. **c** Residual three-dimensional (3D) fluctuating field ***u***_3*D*_ containing small-scale turbulent fluctuations; see Eq. (3) for details. **d** Total energy spectra for the full field (black solid line), the 2D3C component (red squares), and the 3D fluctuations (purple triangles). The shaded gray region indicates the forcing band around *k*_*f*_, and the vertical dashed line marks the Kolmogorov wavenumber *k*_*ν*_. The inset shows the temporal evolution of the total kinetic energy normalized by its stationary mean; the shaded yellow interval denotes the portion of the simulation used to construct the training dataset (see “Methods” for details). **e** Compensated energy spectra *k*^5/3^*E*(*k*) highlighting the plateau associated with inertial-range scaling and the viscous cutoff (inset). **f** Probability density function (PDF) of the vertical vorticity component *ω*_*z*_, normalized by its standard deviation *σ*(*ω*_*z*_), revealing pronounced non-Gaussian tails associated with intermittency. The inset shows a two-dimensional contour of *ω*_*z*_ in a plane perpendicular to the rotation axis, with red and blue indicating intense positive and negative vortical structures, respectively.

We consider rotating turbulence as a paradigmatic benchmark, given its broad relevance in geophysical and industrial applications as well as the difficulty of constructing faithful phenomenological and data-driven surrogates. Rotating turbulence remains an exceptionally difficult problem due to: (i) the coexistence of strong large-scale coherent structures, including cyclonic and anticyclonic vortices, and intense, intermittent small-scale fluctuations in a fully three-dimensional setting (see Fig. 1); and (ii) the system being far from equilibrium, with energy transferred simultaneously toward both large and small scales in a strongly anisotropic manner^32^. In contrast to HIT, where multifractal phenomenology provides a useful statistical framework^17^, no comparable phenomenological description currently exists for the anisotropic, multiscale, and strongly non-Gaussian statistics observed in rotating turbulence.

Motivated by the need to preserve physical consistency in generated fields, related efforts have sought to incorporate physical information into diffusion-based generative frameworks through several strategies. One representative direction augments the diffusion loss with physics-informed penalty terms or residual constraints, as in physics-informed diffusion models^33^. Such approaches can mitigate local violations of governing equations, but act through auxiliary constraint penalties and leave the underlying diffusion training objective unchanged. A complementary line of work enforces constraints during sampling through guidance or projection, including diffusion posterior sampling^34^, training-free guidance^35^, and projected diffusion models^36^. These methods mainly modify the reverse sampling procedure, whereas the present work targets the unconditional synthesis problem, where physically admissible velocity fields must be generated directly from noise. Recent work has also explored enforcing divergence-free constraints within diffusion models for incompressible flows^37^.

In this work, we propose a physics-constrained diffusion model in which fundamental physical constraints are incorporated directly into the generative dynamics. By focusing on incompressibility and zero-mean momentum, the framework explicitly accounts for these a priori constraints of the target velocity-field distribution during both learning and generation. This formulation enables the stable and statistically faithful synthesis of inertial-range three-dimensional turbulent velocity fields at a resolution up to 256^3^ grid points. Our results highlight the importance of incorporating physical constraints directly into the generative dynamics when learning high-dimensional physical systems. In contrast, we show that a direct application of standard diffusion models without such constraints produces velocity fields with multiscale statistical deviations and violations of physical consistency. The proposed framework also leads to substantially more stable and efficient training compared with unconstrained diffusion models.

## Results

We first introduce rotating turbulence as a challenging benchmark for generative modeling and outline the proposed physics-constrained diffusion framework. We then examine spectral accuracy and training convergence, multiscale intermittency, and the physical consistency of the generated fields. Finally, we assess the robustness of the framework across different constraint implementations and examine its sensitivity to training-set size and model capacity. Unless otherwise specified, all statistical quantities reported in the following figures are computed using ensembles of 600 generated flow fields and 600 DNS reference fields, both at the Galerkin-truncated 64^3^ resolution.

### The rotating turbulence challenge

The dataset used for training is extracted from a high-resolution direct numerical simulation (DNS) of rotating turbulence, a paradigmatic 3D turbulent configuration of key importance in many industrial and geophysical contexts^32,38,39^ governed by the incompressible Navier-Stokes equations in a frame with uniform rotation, Ω:

1 $$\dfrac{\partial {{\boldsymbol{u}}}}{\partial t}+{{\boldsymbol{u}}}\cdot \nabla {{\boldsymbol{u}}}+2{{\boldsymbol{\Omega }}}\times {{\boldsymbol{u}}}=-\nabla \tilde{p}+\nu \Delta {{\boldsymbol{u}}}+{{\boldsymbol{f}}}.$$

Details of the numerical setup and data preparation are provided in Methods. The resulting velocity field ***u***(*x*, *y*, *z*, *t*) represents a fully developed turbulent state with strong rotational effects (see Fig. 1a for a 3D rendering), evolving chaotically in time (inset of panel (d)), and posing a fundamental challenge for generative modeling. In particular, it simultaneously involves an extremely large number of active degrees of freedom, organized in quasi-2D coherent vortical structures (panel (b)) and a strongly anisotropic 3D turbulent background (panel (c)), with pronounced multiscale fluctuations as shown by the power-law energy spectra (panels (d,e)) and strong non-Gaussianity evidenced by the presence of fat tails in the distribution of vorticity (panel (f)).

The first source of complexity arises from the high dimensionality of the flow, involving a large number of dynamically active degrees of freedom distributed across scales. This is quantified by the kinetic energy spectrum,

2 $$E(k)=\dfrac{1}{2}{\sum}_{k\le | {{\boldsymbol{k}}}^{\prime} | < k+1}{\sum}_{i\in \{x,y,z\}}| {\hat{u}}_{i}({{\boldsymbol{k}}}^{\prime} ){| }^{2},$$

where ${\hat{u}}_{i}({{\boldsymbol{k}}}^{\prime} )$ denotes the Fourier coefficient of the *i*th velocity component *u*_*i*_(***x***) at wavevector ${{\boldsymbol{k}}}^{\prime}$. As shown in Fig. 1d, the energy spectrum exhibits a power-law behavior, *E*(*k*) ~ *k*^−5/3^, in the wavenumber range *k*_*f*_ < *k* < *k*_*ν*_, bounded by the forcing scale *k*_*f*_ and the viscous cutoff, corresponding to the Kolmogorov wavenumber *k*_*ν*_ ≈ 32. Beyond *k*_*ν*_, the spectrum decays exponentially due to viscous dissipation. As a result, the dynamically relevant scales of the flow are well captured by coarse-graining the data on a cube of size 64^3^.

A second source of complexity stems from the strong anisotropy induced by rotation, manifested by the coexistence of two-dimensional three-component (2D3C) vortical structures in the direction parallel to rotation and a strongly fluctuating 3D background (see Fig. 1b, c). These two components are associated with the slow and fast manifolds, respectively, which are connected to the presence of inverse and direct energy cascades (see ref. ^32^ and references therein). Accordingly, we decompose the velocity field as

3 $${{\boldsymbol{u}}}(x,y,z)={{{\boldsymbol{u}}}}_{2D3C}(x,y)+{{{\boldsymbol{u}}}}_{3D}(x,y,z),$$

where the 2D3C field is defined as the average of the velocity field along the rotation axis,

4 $${{{\boldsymbol{u}}}}_{2D3C}(x,y)=\dfrac{1}{{L}_{0}}\int_{0}^{{L}_{0}}{{\boldsymbol{u}}}(x,y,z)\,dz,$$

where *L*_0_ denotes the domain size. The field ***u***_3*D*_ denotes the residual three-dimensional fluctuating component. The corresponding energy spectra of the 2D3C component, *E*_2*D*3*C*_(*k*), and of the 3D fluctuating contribution, *E*_3*D*_(*k*), shown in Fig. 1d, e, show that the 2D3C velocity components dominate at large scales, while the 3D velocity fluctuations are strongly suppressed and become significant only at smaller scales, signaling the gradual recovery of fully three-dimensional dynamics.

Third, the flow exhibits strong intermittency. This is reflected in the probability density function (PDF) of the vertical vorticity component, *ω*_*z*_ = (∇×***u***)_*z*_, shown in Fig. 1f, which exhibits pronounced non-Gaussian tails associated with intense, spatially localized vortical structures. Beyond single-point statistics, intermittency manifests across scales and is strongly shaped by the anisotropic organization induced by rotation. These effects will be quantified below using scale-dependent flatness based on transverse velocity increments, with separate contributions from the 2D3C and 3D fluctuations.

Beyond these statistical challenges, the velocity field is subject to strict physical constraints, including incompressibility and zero-mean momentum, which must be satisfied exactly and pose additional challenges for any generative modeling approach.

In the present context, we denote each instantaneous three-dimensional turbulent velocity field of the ground truth DNS by

$${{{\mathcal{V}}}}_{0}\equiv \{{{\boldsymbol{u}}}({{\boldsymbol{x}}})\,| \,{{\boldsymbol{x}}}\in {{\mathcal{D}}}\},$$

where ${{\mathcal{D}}}$ is the spatial domain. The velocity field satisfies incompressibility and zero-mean momentum:

5 $$\nabla \cdot {{\boldsymbol{u}}}=0\quad \forall \,{{\boldsymbol{x}}}\in {{\mathcal{D}}},\qquad \langle {{\boldsymbol{u}}}\rangle={{\mathbf{0}}},$$

where 〈 ⋅ 〉 denotes the spatial average over ${{\mathcal{D}}}$.

### Physics-constrained diffusion framework

**Fig. 2:**
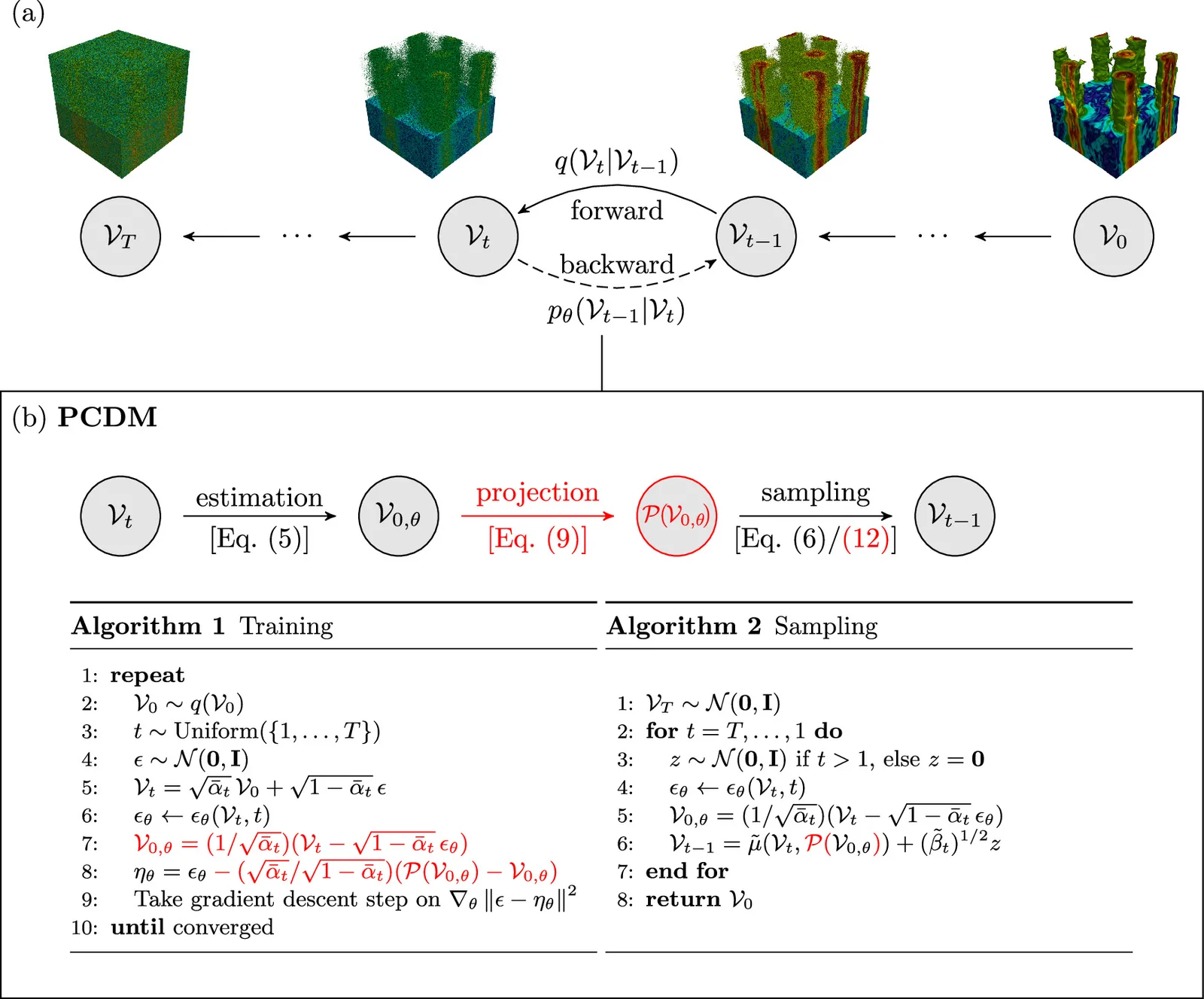
Diffusion framework with physics-constrained backward dynamics (PCDM). **a** Forward and backward diffusion processes for a 3D turbulent velocity field. The forward process $q({{{\mathcal{V}}}}_{t}| {{{\mathcal{V}}}}_{t-1})$ progressively injects Gaussian noise until the distribution approaches an isotropic Gaussian at ${{{\mathcal{V}}}}_{T}$, while the learned backward process ${p}_{\theta }({{{\mathcal{V}}}}_{t-1}| {{{\mathcal{V}}}}_{t})$ reverses this procedure to generate physically consistent velocity fields. **b** Physics-constrained backward transition in the PCDM. The neural network predicts the noise *ϵ*_*θ*_, from which a clean estimate for the final generative step *t* = 0, ${{{\mathcal{V}}}}_{0,\theta }$, is obtained. This estimated 3D field is projected onto the physically admissible subspace, enforcing incompressibility and zero-mean momentum, yielding ${\tilde{{{\mathcal{V}}}}}_{0,\theta }$. A sample ${{{\mathcal{V}}}}_{t-1}$ is then drawn from the Gaussian distribution defined by the reverse transition in Eq. (22), and the procedure is repeated until the final sample ${{{\mathcal{V}}}}_{0}$ is obtained. The pseudocode below summarizes the training and sampling procedures of the PCDM. Removing the projection-based correction (highlighted in red in both the schematic and the algorithms) reduces the method to the standard DDPM formulation.

To synthesize turbulent velocity fields subject to these constraints, we employ a physics-constrained diffusion model (PCDM), obtained by modifying the standard denoising diffusion probabilistic model (DDPM)^18,40^. As illustrated schematically in Fig. 2, the clean-field estimate predicted at each reverse-diffusion step is projected onto the physically admissible subspace, and the resulting correction is incorporated into both the training objective and the generative process. Removing this correction reduces the framework to the standard DDPM formulation. Specifically, incompressibility and zero-mean momentum can be enforced exactly by projecting the velocity field in Fourier space as

6 $$\widehat{{{\boldsymbol{u}}}}({{\boldsymbol{k}}})\to \left({{\bf{I}}}-\dfrac{{{{\boldsymbol{kk}}}}^{\top }}{| {{\boldsymbol{k}}}{| }^{2}}\right)\widehat{{{\boldsymbol{u}}}}({{\boldsymbol{k}}}),\qquad \widehat{{{\boldsymbol{u}}}}({{\mathbf{0}}})\to {{\mathbf{0}}},$$

where $\widehat{(\cdot )}$ denotes the Fourier transform and ***k*** is the wavevector. We denote the projection of a generic 3D velocity field configuration ${{{\mathcal{V}}}}_{0}$ using Eq. (6) by ${{\mathcal{P}}}({{{\mathcal{V}}}}_{0})$. Different implementations of these physical constraints are compared below. The complete PCDM formulation is provided in Methods.

### Spectral accuracy and training convergence

To assess the performance of the proposed framework, we compare the PCDM with two baseline approaches: (i) a standard DDPM implementation, corresponding to the scheme of Fig. 2 without the physics-constrained operations highlighted in red, referred to as DDPM-std; and (ii) a progressively trained variant of the DDPM (DDPM-prog), designed to alleviate the optimization difficulty associated with the extremely high dimensionality of the full 3D velocity field (see “Methods”).

**Fig. 3:**
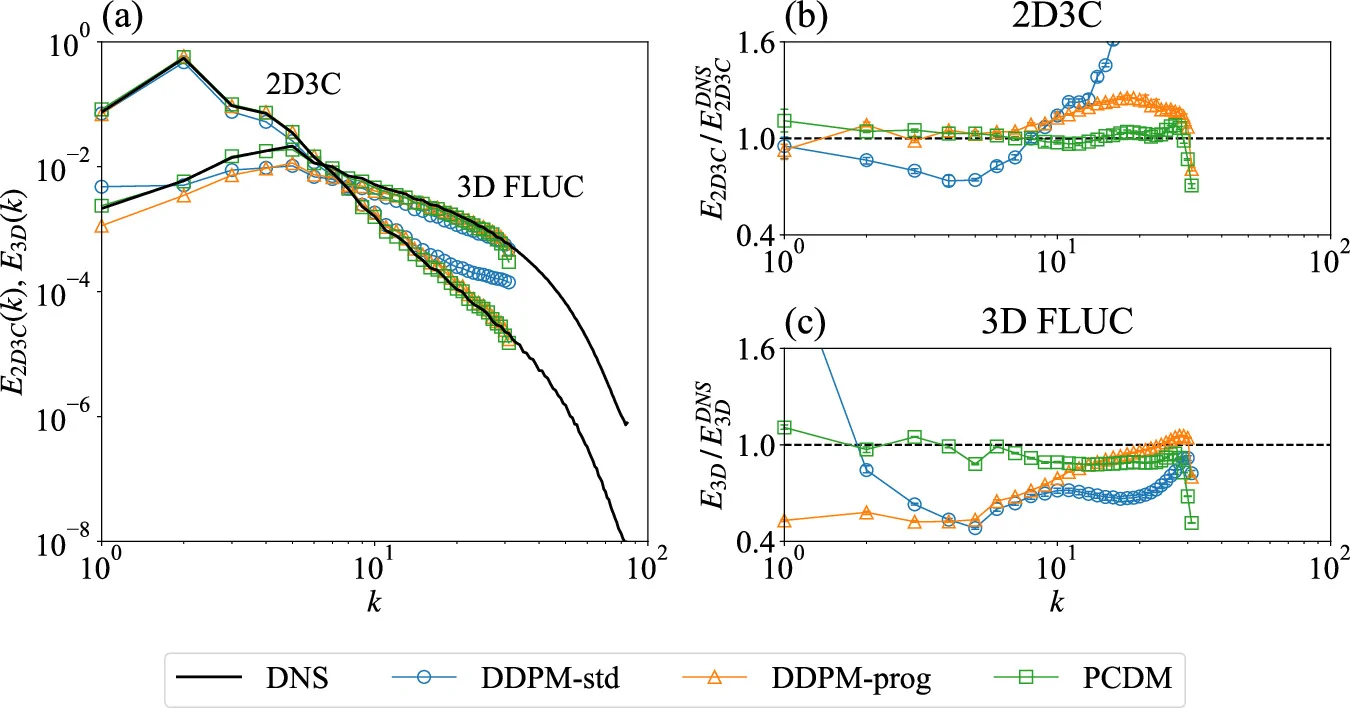
Multiscale spectral diagnostics for DDPM-std, DDPM-prog, and the PCDM. Here, DDPM-std and DDPM-prog denote the standard and progressively trained variants, respectively. **a** Energy spectra of the 2D3C component and the three-dimensional fluctuating field (3D FLUC), with the DNS reference (black solid line). **b** Ratio of generated to DNS spectra for the 2D3C component. **c** Same as (**b**), but for the 3D fluctuating component. Error bars are of the order of the symbol size and indicate variability estimated over three independent batches, each consisting of 200 generated fields.

Figure 3a shows that the PCDM reproduces the target energy spectra with high fidelity, matching the DNS reference across all scales for both the 2D3C and the 3D components. The spectral ratios shown in panels (b,c) demonstrate a clear advantage of the PCDM over the two baseline approaches. DDPM-std exhibits pronounced scale-dependent deviations, while DDPM-prog improves the agreement with DNS but retains noticeable deviations over a broad range of scales. In contrast, the PCDM provides the closest agreement with the DNS spectra across nearly the entire resolved range of scales.

**Fig. 4:**
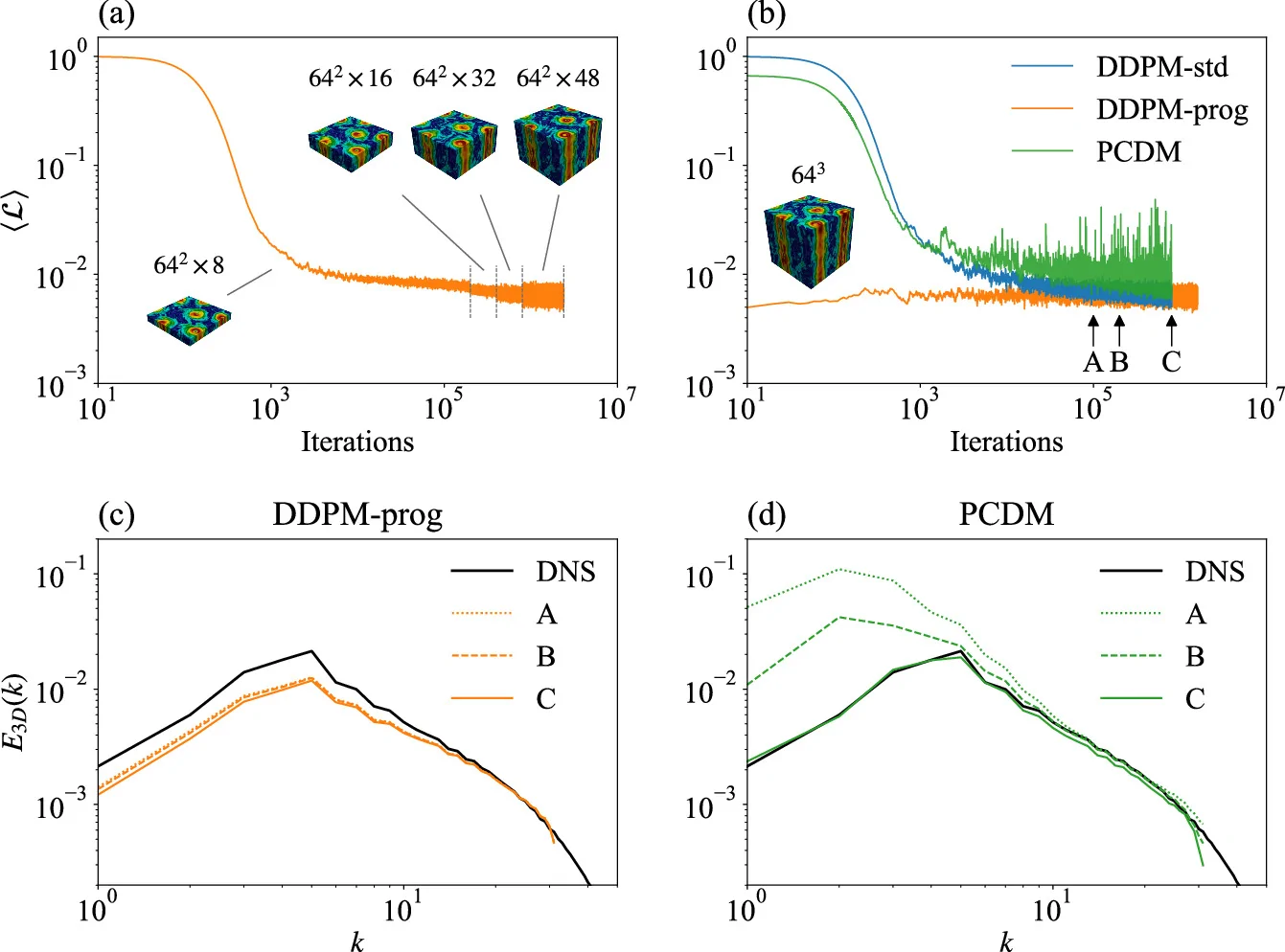
Training dynamics and convergence. **a** Pretraining loss of DDPM-prog, where the input domain is expanded from a thin slab toward progressively thicker domains. **b** Training loss of DDPM-std, DDPM-prog, and the PCDM on the full 64^3^ dataset. Three representative iterations (100 k, 200 k, and 800 k), labeled A, B, and C, are selected from panel (**b**) to assess convergence. **c** Energy spectra of the 3D fluctuating component (3D FLUC) generated by DDPM-prog at iterations A, B, and C, compared with the DNS reference (black). **d** Same as (**c**), but for the PCDM. While both models exhibit progressive convergence during training, only the PCDM converges to the DNS spectrum across scales.

Notably, Fig. 4 highlights the superior training behavior of the PCDM compared with the two baseline approaches. In addition to its improved spectral accuracy, the PCDM converges significantly faster than the unconstrained DDPMs. In practice, the PCDM requires approximately 12 days of training on four NVIDIA A100 GPUs, corresponding to a roughly five-fold reduction in training time relative to DDPM-prog (see Methods for further details on the computational cost). Panel (a) shows the pretraining stage of DDPM-prog, where the input domain is gradually expanded from a thin slab toward progressively thicker three-dimensional domains (see Methods). Panel (b) reports the training loss of DDPM-std, DDPM-prog, and the PCDM during training on the full 64^3^ dataset. Here $\langle {{\mathcal{L}}}\rangle$ denotes the batch-averaged diffusion training loss defined in Eq. (23). To further assess convergence of the generated fields, three representative training iterations labeled A, B, and C are selected from panel (b). Panels (c) and (d) report the corresponding energy spectra of the 3D fluctuating component generated by DDPM-prog and the PCDM, respectively. While DDPM-prog stabilizes after a long pretraining phase, it does not fully recover the DNS spectral behavior across scales. In contrast, the PCDM progressively converges toward the DNS spectrum during training.

For the 64^3^ fields considered here, generating 640 independent samples with any of the trained models requires approximately 2 hours on four NVIDIA A100 GPUs. For context, collecting the 600 statistically decorrelated DNS snapshots used in this work required approximately 112 wall-clock hours on 64 CPU cores (see “Methods”). This comparison is not one-to-one: DNS evolves temporally correlated, dynamically consistent trajectories and resolves the full range of dynamically relevant scales, whereas the trained diffusion model samples independent single-snapshot fields from the learned single-time distribution over the retained inertial-range scales.

### Intermittency and multiscale statistical fidelity

Following previous studies^41^, we characterize multiscale deviations from Gaussian statistics through the analysis of transverse velocity increments measured in the plane normal to the rotation axis,

7 $$\delta {u}_{\perp }({{\boldsymbol{r}}})=\left[{{\boldsymbol{u}}}({{\boldsymbol{x}}}+{{\boldsymbol{r}}})-{{\boldsymbol{u}}}({{\boldsymbol{x}}})\right]\cdot \hat{{{\boldsymbol{t}}}},$$

where the separation vector ***r*** lies in this plane and $\hat{{{\boldsymbol{t}}}}$ is orthogonal to both ***r*** and the rotation axis. Assuming isotropy within the plane normal to the rotation axis, the fourth-order flatness is defined as

8 $${K}_{\perp }^{(4)}(r)=\dfrac{\langle {[\delta {u}_{\perp }(r)]}^{4}\rangle }{{\langle {[\delta {u}_{\perp }(r)]}^{2}\rangle }^{2}},$$

with larger values indicating stronger intermittency.

**Fig. 5:**
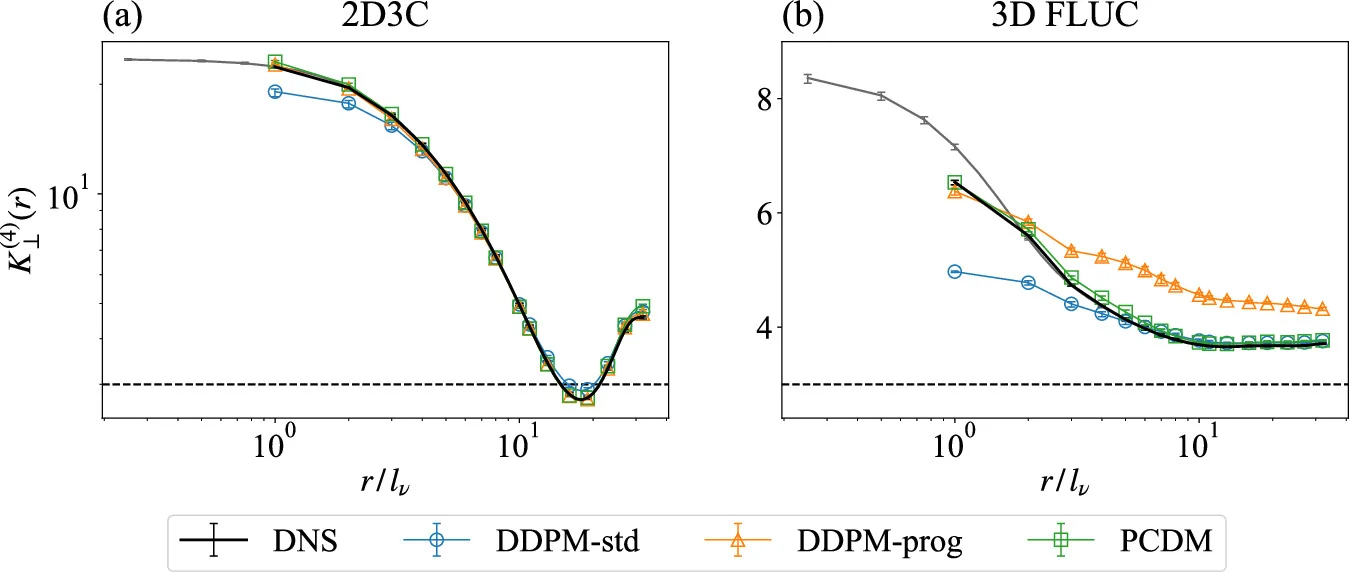
Scale-dependent fourth-order flatness. Fourth-order flatness ${K}_{\perp }^{(4)}(r)$ of transverse velocity increments, shown as a function of the separation *r*/*l*_*ν*_, where *l*_*ν*_ denotes the Kolmogorov scale, measured in the plane normal to the rotation axis, for the reference DNS (64^3^), DDPM-std, DDPM-prog, and the PCDM. Results are reported separately for (**a**) the slow 2D3C component and (**b**) the fast, fully three-dimensional fluctuating component (3D FLUC). The dashed horizontal line indicates the Gaussian reference value. The gray curve corresponds to the full-resolution DNS data (256^3^; see “Methods”).

Figure 5 shows the scale-dependent fourth-order flatness of the 2D3C and 3D fluctuating components for the reference DNS and the generated fields. All statistics are evaluated at separations *r*/*l*_*ν*_ within the inertial-range scales resolved in the 64^3^ data, where *l*_*ν*_ = *L*_0_/*k*_*ν*_ is the Kolmogorov length scale. As shown, DDPM-std exhibits systematic, scale-dependent discrepancies in both components: for the 2D3C field, the flatness is underestimated at small scales and overestimated in the intermediate-scale dip, while for the 3D fluctuating component, the flatness is captured at large scales but systematically underestimated at small separations. Progressive training (DDPM-prog) improves the agreement for the 2D3C component, yielding a flatness profile close to the DNS reference, consistent with the staged exposure of the model to increasing complexity along the rotation axis. In contrast, for the 3D fluctuating component, DDPM-prog exhibits large discrepancies across all scales, indicating that the 3D statistics are not captured by progressive training starting from thin quasi-2D slabs. Conversely, our PCDM accurately reproduces the DNS flatness for both the 2D3C and 3D components across all scales. This result shows that the PCDM-generated fields correctly reproduce the intermittent features of both the slow and fast components in rotating turbulence.

**Fig. 6:**
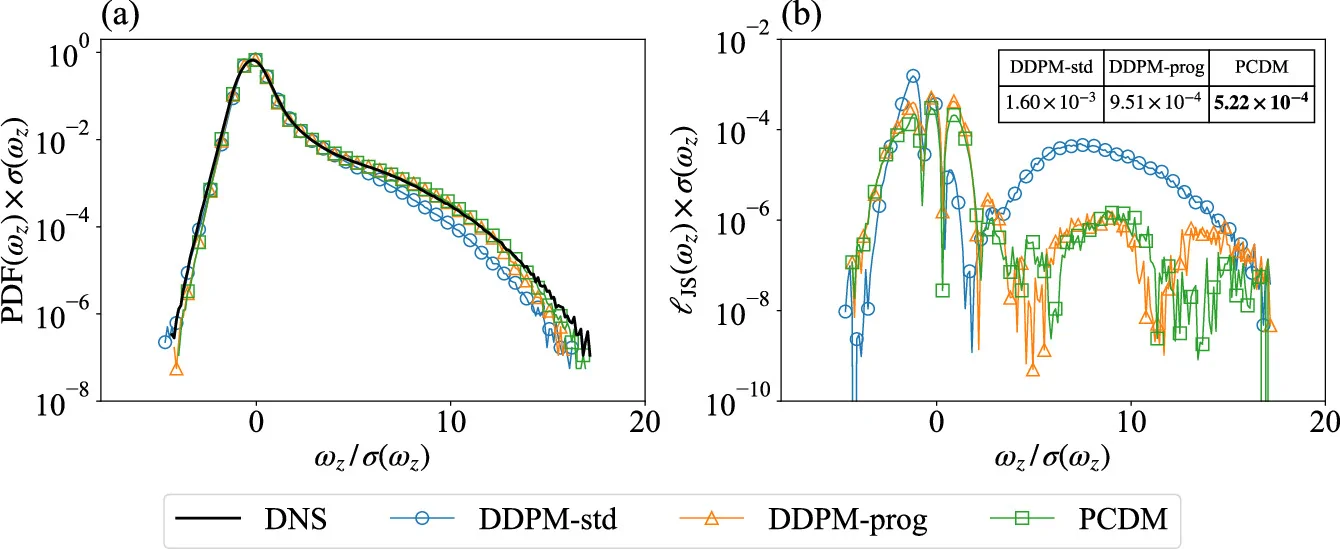
Single-point vorticity statistics. **a** PDFs of the vertical vorticity component *ω*_*z*_ for DNS, DDPM-std, DDPM-prog, and the PCDM, normalized by the DNS standard deviation *σ*(*ω*_*z*_). **b** Local contribution *ℓ*_JS_ to the Jensen--Shannon divergence (JSD) between the model PDFs and the DNS PDF. The inset table reports the integrated JSD *D*_JS_.

Next, we assess the statistical fidelity of the generated fields using single-point vorticity statistics. Figure 6a compares the PDFs of the vertical vorticity component *ω*_*z*_ for DNS and the generated fields. DDPM-std exhibits pronounced deviations in the distribution tails, systematically underestimating extreme vorticity events. In contrast, both DDPM-prog and PCDM capture the heavy-tailed distributions characteristic of intermittent turbulence and show good agreement with the DNS. To quantify the differences between the PDFs, Fig. 6b reports the local contribution to the Jensen–Shannon divergence (JSD) between the model PDFs *p*(*x*) and the DNS PDF *q*(*x*), defined as

9 $${\ell }_{{{\rm{JS}}}}(p(x)\parallel q(x))=\dfrac{1}{2}p(x)\log \dfrac{p(x)}{m(x)}+\dfrac{1}{2}q(x)\log \dfrac{q(x)}{m(x)},$$

where $m(x)=\frac{1}{2}[p(x)+q(x)]$. The JSD is then obtained as

10 $${D}_{{{\rm{JS}}}}(p\!\!\parallel\!\! q)=\int{\ell }_{{{\rm{JS}}}}(p(x)\!\!\parallel\!\! q(x))\,dx.$$

The divergence satisfies $0\le {D}_{{{\rm{JS}}}}\le \ln 2$, where smaller values indicate better agreement between the distributions. The inset table in Fig. 6b reports the integrated JSD values for the different models. PCDM yields the smallest divergence, indicating the closest agreement with the DNS statistics.

### Divergence-free validation

**Fig. 7:**
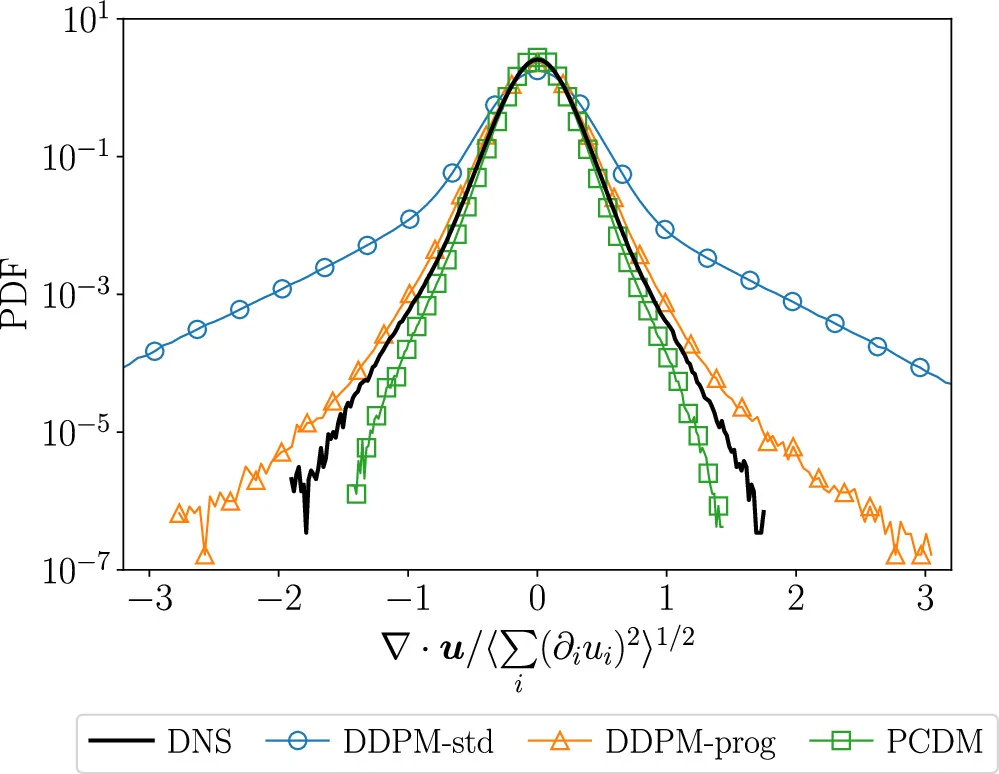
Normalized velocity divergence probability density function. PDF of the normalized velocity divergence for DNS, DDPM-std, DDPM-prog, and the PCDM, computed using second-order finite differences on the Galerkin-truncated 64^3^ fields.

The velocity fields generated by the PCDM are divergence-free at machine precision by construction, as a direct consequence of the physics-constrained generative dynamics. To quantify this property in a fair way, we compute a coarse-grained approximation of the gradients using second-order finite differences on the Galerkin-truncated 64^3^ fields and compare the resulting divergence statistics among the three generative models considered here. Since this finite-difference diagnostic differs from the Fourier-space incompressibility constraint, even DNS and PCDM exhibit a finite discretization-level divergence. In Fig. 7, we show the PDF of the normalized velocity divergence,

$$\nabla \cdot {{\boldsymbol{u}}}/{\langle {\sum}_{i}{({\partial }_{i}{u}_{i})}^{2}\rangle }^{1/2}.$$

DDPM-std exhibits substantially broader tails, indicating clear compressibility errors. DDPM-prog is much closer to the DNS reference, suggesting that progressive training can partially learn an approximately incompressible structure from the training data, although slightly broader tails remain. By contrast, PCDM agrees with the DNS up to the discretization accuracy of this diagnostic. The zero-mean momentum constraint is also explicitly enforced in the PCDM but not in the unconstrained DDPMs.

### Robustness across constraint implementations

We next investigate the robustness of the PCDM framework. Specifically, we examine whether distinct, non-equivalent implementations of the same physical constraints give rise to similar generative behavior, thereby assessing convergence toward a consistent underlying physical representation.

To this end, we consider two variants of the PCDM. The first, denoted *PCDM-Fourier*, is the formulation used throughout the preceding results and enforces incompressibility and zero-mean flow exactly through the Fourier-space projection operator defined in Eq. (6).

The second variant, referred to as *PCDM-Integral*, employs a relaxed formulation of the same physical constraints. Instead of enforcing the exact pointwise incompressibility and zero-mean constraints via the Fourier-space projection, we impose only a necessary integral condition, requiring that the plane-integrated velocity components vanish in each coordinate direction:

$$\int{u}_{i}\,{{\rm{d}}}{x}_{j}\,{{\rm{d}}}{x}_{k}=0,$$

for the three cyclic permutations (*i*, *j*, *k*) = (*x*, *y*, *z*), (*y*, *z*, *x*), and (*z*, *x*, *y*). This condition is weaker than the exact constraint in Eq. (5); a field may satisfy it without being divergence-free at every point. Accordingly, the Fourier-space projector ${{\mathcal{P}}}$ is replaced by an integral projector, ${{{\mathcal{P}}}}_{{{\rm{int}}}}$, which enforces the above condition within the diffusion-based generative process.

Because the reverse process in Eq. (22) is stochastic, we fix the random seed to generate paired velocity-field realizations from PCDM-Fourier and PCDM-Integral using identical noise histories:

$${{{\mathcal{V}}}}^{(F)}\equiv \{{{{\boldsymbol{u}}}}^{(F)}({{\boldsymbol{x}}})\,| \,{{\boldsymbol{x}}}\in {{\mathcal{D}}}\},\quad {{{\mathcal{V}}}}^{(I)}\equiv \{{{{\boldsymbol{u}}}}^{(I)}({{\boldsymbol{x}}})\,| \,{{\boldsymbol{x}}}\in {{\mathcal{D}}}\}.$$

We then quantify the similarity between paired realizations using the cosine similarity:

11 $${{\mathcal{C}}}=\dfrac{\langle {{{\boldsymbol{u}}}}^{(F)}({{\boldsymbol{x}}})\cdot {{{\boldsymbol{u}}}}^{(I)}({{\boldsymbol{x}}})\rangle }{{\langle | {{{\boldsymbol{u}}}}^{(F)}({{\boldsymbol{x}}}){| }^{2}\rangle }^{1/2}{\langle | {{{\boldsymbol{u}}}}^{(I)}({{\boldsymbol{x}}}){| }^{2}\rangle }^{1/2}},$$

where 〈 ⋅ 〉 denotes spatial averaging.

**Fig. 8:**
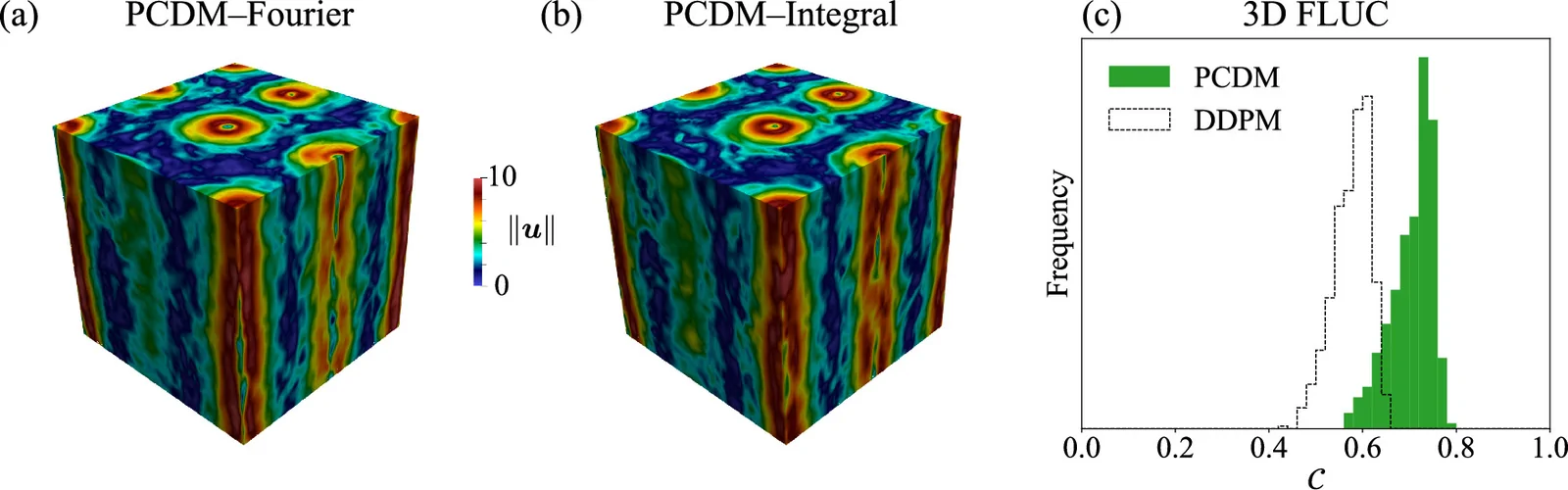
Robustness of physics-constrained diffusion models under different constraint implementations. Paired samples are generated using identical noise histories during the backward diffusion process. **a** Velocity magnitude of the full velocity field (dominated by the 2D3C component) generated using the Fourier-based constraint formulation (PCDM-Fourier). **b** Corresponding field generated using the relaxed integral constraint formulation (PCDM-Integral). The two realizations display similar large-scale, 2D3C-dominated structures, while visible differences remain. **c** Histogram of the cosine similarity ${{\mathcal{C}}}$ computed for the three-dimensional fluctuating component (3D FLUC) between paired samples. In panel (**c**), the legend entry “PCDM” refers to the similarity between PCDM-Fourier and PCDM-Integral, while “DDPM” refers to the similarity between DDPM-prog and PCDM-Fourier (shown for reference). The high similarity values indicate that the two PCDM variants generate highly correlated realizations even for the fully 3D fluctuations.

A representative pair of velocity fields generated by the two models is shown in Fig. 8a, b. The realizations display very similar large-scale structures, dominated by the 2D3C component, although small-scale differences are visible. To quantify the similarity between the two PCDM variants, we compute the cosine similarity ${{\mathcal{C}}}$ for the 3D fluctuating component, which represents the most challenging part of the flow. While PCDM captures this component well, DDPM-prog fails to do so. The resulting distribution of ${{\mathcal{C}}}$ is shown in Fig. 8c. Despite the physical non-equivalence of the two constraint formulations, the cosine similarity remains high, indicating that the two PCDM variants generate strongly correlated realizations when driven by the same stochastic noise. This demonstrates that the PCDM framework is robust with respect to different implementations of the physical constraints and converges toward a consistent physical representation.

### Sensitivity to training-set size and model capacity

To assess whether the high statistical fidelity of the PCDM-generated fields reflects learning of the underlying turbulent distribution rather than direct memorization of the training data, we perform a nearest-neighbor cosine-similarity analysis. The generated fields exhibit a similarity distribution comparable to that of held-out DNS fields, providing no evidence of direct training-sample memorization under this metric (see Supplementary Fig. 1).

We further assess the sensitivity of the generated statistics to training-set size and model capacity (Supplementary Figs. 2, 3). Reducing the training-set size or model capacity leads to larger deviations from the DNS statistics, particularly for the weaker 3D fluctuating component, whereas the dominant 2D3C component remains comparatively robust. Increasing the capacity beyond the reference model does not yield systematic improvements despite the additional computational cost.

## Discussion

In this work, we have shown that a direct application of standard denoising diffusion probabilistic models to fully developed three-dimensional turbulence does not achieve statistically faithful generation of velocity fields, even in the simplest single-snapshot setting. The generated samples exhibit pronounced scale-dependent deviations in anisotropic energy spectra and intermittency statistics, together with systematic violations of fundamental physical constraints, including incompressibility and zero-mean momentum. These failures persist despite apparent training convergence and the use of progressive training strategies, suggesting that they cannot be resolved by optimization improvements alone in systems with extremely large numbers of strongly coupled degrees of freedom.

This behavior points to a more fundamental limitation of standard diffusion training objectives when applied to complex physical systems governed by global constraints. Because the denoising objective is blind to constraints such as incompressibility and zero-mean momentum, the learned distribution is not properly confined to physically admissible configurations, rendering modeling in such high-dimensional settings intrinsically fragile.

To address this limitation, we introduced a physics-constrained diffusion model (PCDM) designed to incorporate a priori physical constraints directly into the learned generative distribution. We demonstrated the proposed approach to solve an open problem of interest for the community working on geophysical fluid dynamics, providing a stochastic generative modeling framework for 3D rotating turbulence that strictly satisfies the imposed physical constraints, while accurately reproducing anisotropic energy spectra and multiscale intermittency statistics, in close agreement with the DNS reference.

Our results open the way to using generative models as physically consistent probabilistic priors for single-snapshot 3D turbulent fields. Such priors may be useful for quality estimation and for data assimilation through guided sampling conditioned on observations, both of which are relevant in geophysical, environmental, and astrophysical contexts^42–47^. In addition, such priors could also support the statistical exploration of rare and extreme events in high-dimensional multiscale systems^48^.

Although our work represents one of the first applications of generative diffusion models to fully 3D turbulence – addressing important bottlenecks such as intermittency and multiscale structure, which are absent in 2D flows – much work remains ahead, both technologically and conceptually, before data-driven approaches can rival the capacity of the Navier-Stokes equations to generate fully developed turbulence at high resolution. For example, to date, no data-driven model is capable of generating three-dimensional turbulent flows at Reynolds numbers high enough for exascale applications. Such regimes require simulations at resolutions of billions (or even trillions) of grid points, with fully three-dimensional turbulent velocity fields evolved over long time horizons. We currently lack both high-quantity, high-quality ground-truth datasets for training and the necessary computational resources to achieve this goal. More generally, a systematic characterization of the interplay between model capacity, training-set size, and the complexity of the underlying turbulent system remains an important open problem. From a physical perspective, this can be viewed as a phase diagram spanned by these quantities, with regimes corresponding to generalization, memorization, and model failure^49–52^. Such a phase diagram would be valuable for guiding practical applications of generative models to turbulence and for understanding their behavior as the complexity of the target flow increases. This issue is particularly relevant to fully developed turbulence, where the dimension of the turbulent attractor grows as *Re*^9/4^, with *Re* the Reynolds number and a direct measure of the turbulent intensity^17^.

Beyond the specific setting considered here, our results point to a broader implication for diffusion-based generative modeling of complex physical systems. In systems where admissible configurations are strongly constrained by physical laws, faithful generative modeling benefits from incorporating such constraints directly into the generative dynamics. Physics-constrained diffusion models thus offer a natural route toward aligning learned distributions with the structure of the physically admissible configuration space. In this context, the present PCDM framework naturally invites extensions to other incompressible flow configurations, including wall-bounded turbulence with boundary-condition and flux constraints, as well as incompressible magnetohydrodynamics, where the velocity and magnetic fields must both remain divergence-free. In principle, temporal information could be incorporated by extending the generation target from a single velocity field to a short sequence of velocity fields and adopting a network architecture capable of modeling temporal correlations. This would naturally increase the computational cost, since multiple three-dimensional fields would need to be processed. However, the current formulation is tailored to constraints that act at the level of individual realizations and admit an explicit projection onto the admissible space. Extending this approach to nonlinear, statistical, or approximate constraints, as well as to temporal consistency across successive flow states, remains an important challenge for future work.

## Methods

In this section, we describe the direct numerical simulation and the preparation of the training dataset, review the standard denoising diffusion probabilistic model (DDPM) formulation^18,40^, and present the physics-constrained diffusion model (PCDM) for synthesizing fully developed 3D turbulent velocity fields. The PCDM is obtained by a suitable modification of the DDPM framework. We also report the implementation and training details.

### Direct numerical simulation

We consider statistically stationary rotating turbulence governed by the three-dimensional incompressible Navier-Stokes equations in a periodic domain with uniform rotation,

12 $$\dfrac{\partial {{\boldsymbol{u}}}}{\partial t}+{{\boldsymbol{u}}}\cdot \nabla {{\boldsymbol{u}}}+2{{\boldsymbol{\Omega }}}\times {{\boldsymbol{u}}}=-\nabla \tilde{p}+\nu \Delta {{\boldsymbol{u}}}+{{\boldsymbol{f}}},$$

where ***u*** is the 3D velocity field, $\tilde{p}$ is the modified pressure in the rotating frame enforcing incompressibility,  ∇ ⋅ ***u*** = 0, and ${{\boldsymbol{\Omega }}}={{\Omega }}\hat{{{\boldsymbol{z}}}}$ is the rotation vector. The flow is simulated using DNS with a pseudo-spectral method on a 256^3^ grid. A large-scale stochastic forcing ***f*** injects energy near the wavenumber *k*_*f*_ = 4, implemented as a second-order Ornstein-Uhlenbeck process^53,54^. The forcing scale is chosen to allow both forward and inverse energy cascades. To extend the inertial range, the viscous term *ν* Δ ***u*** is replaced by a hyperviscous dissipation *ν*_*h*_ ∇^4^ ***u***, and a linear large-scale friction *β*_*f*_ Δ^−1^***u*** is applied to suppress the formation of a large-scale condensate^32^. The flow reaches a statistically stationary regime characterized by a Rossby number $Ro={{{\mathcal{E}}}}^{1/2}/({{\Omega }}/{k}_{f})\approx 0.1$, where ${{\mathcal{E}}}=\langle | {{\boldsymbol{u}}}{| }^{2}\rangle /2$ is the kinetic energy. The integral length scale is defined as *L* = *L*_0_/*k*_*f*_ ≈ 0.25*L*_0_, and the corresponding eddy turnover time is ${T}_{L}=L/\sqrt{2{{\mathcal{E}}}}\approx 0.43$, where *L*_0_ = 2*π* is the domain size. Further details of the simulation are provided in ref. ^55^.

### Preparation of the training dataset

To enable generative modeling of 3D turbulent flow fields at single time instances, we construct a training dataset consisting of velocity snapshots sampled from the statistically stationary regime of the flow. A total of 600 snapshots are collected with a temporal spacing of Δ*t*_*s*_ ≈ 2.34*T*_*L*_ between successive samples to ensure statistical decorrelation, requiring approximately 112 wall-clock hours on 64 CPU cores, excluding the initial transient needed to reach the statistically stationary regime (see the inset of Fig. 1d). The resolution of the sampled fields is then reduced from 256^3^ to 64^3^ by applying a Galerkin truncation in Fourier space,

13 $${{\boldsymbol{u}}}({{\boldsymbol{x}}})={\sum }_{\parallel {{\boldsymbol{k}}}\parallel \le {k}_{\nu }}\hat{{{\boldsymbol{u}}}}({{\boldsymbol{k}}})\,{{{\rm{e}}}}^{{{\rm{i}}}{{\boldsymbol{k}}}\cdot {{\boldsymbol{x}}}},$$

where the cutoff wavenumber is set to the viscous Kolmogorov scale *k*_*ν*_ ≈ 32. This truncation removes only fully dissipative Fourier modes, corresponding to scales at which the flow dynamics are nearly linear, while retaining the entire inertial range and its nonlinear interactions. As a result, the reduced-resolution fields preserve all essential multiscale, anisotropic, and intermittent features of the flow, as well as the exact physical constraints of incompressibility and zero-mean momentum, while providing a computationally tractable representation for generative modeling.

### Denoising diffusion probabilistic model

In the present setting, we aim to learn the distribution of instantaneous turbulent flow states, $q({{{\mathcal{V}}}}_{0})$, from DNS snapshots and to sample new synthetic velocity fields from the learned distribution. The DDPM framework consists of a pair of Markov processes, as illustrated schematically in Fig. 2a: a fixed forward diffusion process, $q({{{\mathcal{V}}}}_{t}| {{{\mathcal{V}}}}_{t-1})$, that progressively corrupts the data with noise, and a learned reverse process, ${p}_{\theta }({{{\mathcal{V}}}}_{t-1}| {{{\mathcal{V}}}}_{t})$, that aims to recover the data starting from independent Gaussian samples. The forward process generates a sequence of increasingly noisy latent variables ${{{\mathcal{V}}}}_{1},\ldots,{{{\mathcal{V}}}}_{T}$ by injecting Gaussian noise into a clean sample ${{{\mathcal{V}}}}_{0} \sim q({{{\mathcal{V}}}}_{0})$ according to

14 $${{{\mathcal{V}}}}_{t}=\sqrt{{\bar{\alpha }}_{t}}\,{{{\mathcal{V}}}}_{0}+\sqrt{1-{\bar{\alpha }}_{t}}\,\epsilon,\qquad \epsilon \sim {{\mathcal{N}}}({{\mathbf{0}}},{{\bf{I}}}),$$

where ${\bar{\alpha }}_{t}=\mathop{\prod }_{i=1}^{t}(1-{\beta }_{i})$ is defined in terms of a prescribed noise schedule ${\{{\beta }_{t}\}}_{t=1}^{T}$ (see Supplementary Methods for details). For a sufficiently large *T* and an appropriate noise schedule, the distribution of the terminal state ${{{\mathcal{V}}}}_{T}$ approaches an isotropic Gaussian, $q({{{\mathcal{V}}}}_{T})\approx {{\mathcal{N}}}({{\mathbf{0}}},{{\bf{I}}})$.

The reverse (or backward) generative process aims to sample new data ${{{\mathcal{V}}}}_{0}$ starting from Gaussian noise through the learned reverse transitions ${p}_{\theta }({{{\mathcal{V}}}}_{t-1}| {{{\mathcal{V}}}}_{t})$ parameterized by a neural network. DDPMs train the neural network to predict the Gaussian noise added during the forward diffusion process, ${\epsilon }_{\theta }({{{\mathcal{V}}}}_{t},t)\approx \epsilon$, yielding at each time step an estimate of the clean field

15 $${{{\mathcal{V}}}}_{0,\theta }({{{\mathcal{V}}}}_{t},t)=\dfrac{1}{\sqrt{{\bar{\alpha }}_{t}}}\left({{{\mathcal{V}}}}_{t}-\sqrt{1-{\bar{\alpha }}_{t}}\,{\epsilon }_{\theta }({{{\mathcal{V}}}}_{t},t)\right).$$

Following^18^, the reverse transition is parameterized as a learnable Gaussian distribution:

16 $${p}_{\theta }({{{\mathcal{V}}}}_{t-1}| {{{\mathcal{V}}}}_{t})={{\mathcal{N}}}\left({{{\mathcal{V}}}}_{t-1};{\tilde{\mu }}_{t}({{{\mathcal{V}}}}_{t},{{{\mathcal{V}}}}_{0,\theta }),{\tilde{\beta }}_{t}{{\bf{I}}}\right),$$

where the mean ${\tilde{\mu }}_{t}$ and the variance ${\tilde{\beta }}_{t}$ are explicitly defined in Supplementary Methods.

### Physics-constrained diffusion model

While standard DDPMs learn the distribution $q({{{\mathcal{V}}}}_{0})$ in a purely data-driven manner, the velocity fields considered here are known a priori to satisfy the constraints in Eq. (5). Within the reverse diffusion dynamics, the network predicts a clean-field estimator ${{{\mathcal{V}}}}_{0,\theta }$ defined in Eq. (15), corresponding to the posterior mean of the clean velocity field conditioned on the noisy state ${{{\mathcal{V}}}}_{t}$:

17 $${{{\mathcal{V}}}}_{0,\theta }\approx {\mathbb{E}}[{{{\mathcal{V}}}}_{0}| {{{\mathcal{V}}}}_{t}]=\int{{{\mathcal{V}}}}_{0}\,p({{{\mathcal{V}}}}_{0}| {{{\mathcal{V}}}}_{t})\,d{{{\mathcal{V}}}}_{0},$$

as implied by Tweedie’s formula for Gaussian denoising^34^. The estimate ${{{\mathcal{V}}}}_{0,\theta }$ is not necessarily compliant with the constraints satisfied by the ground-truth velocity fields, whereas the latter satisfy the relations

18 $${\mathbb{E}}[{{{\mathcal{V}}}}_{0}| {{{\mathcal{V}}}}_{t}]={\mathbb{E}}[{{\mathcal{P}}}({{{\mathcal{V}}}}_{0})| {{{\mathcal{V}}}}_{t}]={{\mathcal{P}}}\,\left({\mathbb{E}}[{{{\mathcal{V}}}}_{0}| {{{\mathcal{V}}}}_{t}]\right).$$

To enforce condition (18) for the estimator in Eq. (17), we project it onto the physically constrained manifold:

19 $${{{\mathcal{V}}}}_{0,\theta }\to {{\mathcal{P}}}({{{\mathcal{V}}}}_{0,\theta }).$$

The velocity field is first transformed to Fourier space, where the projection defined in Eq. (6) is applied, followed by an inverse Fourier transform back to physical space. Replacing ${{{\mathcal{V}}}}_{0,\theta }$ with its projected counterpart ${{\mathcal{P}}}({{{\mathcal{V}}}}_{0,\theta })$ in Eq. (15) yields a corrected noise term *η*_*θ*_:

20 $${{\mathcal{P}}}({{{\mathcal{V}}}}_{0,\theta })=\dfrac{1}{\sqrt{{\bar{\alpha }}_{t}}}\left({{{\mathcal{V}}}}_{t}-\sqrt{1-{\bar{\alpha }}_{t}}\,{\eta }_{\theta }({{{\mathcal{V}}}}_{t},t)\right),$$

given by

21 $${\eta }_{\theta }={\epsilon }_{\theta }-\dfrac{\sqrt{{\bar{\alpha }}_{t}}}{\sqrt{1-{\bar{\alpha }}_{t}}}\left({{\mathcal{P}}}({{{\mathcal{V}}}}_{0,\theta })-{{{\mathcal{V}}}}_{0,\theta }\right).$$

Note that in Eq. (20) neither the noisy realization ${{{\mathcal{V}}}}_{t}$ nor the noise *η*_*θ*_ are required to satisfy the physical constraints, whereas the posterior estimate of the clean velocity field ${{\mathcal{P}}}({{{\mathcal{V}}}}_{0,\theta })$ satisfies both incompressibility and zero-mean momentum. In this way we enforce consistency with the physical constraints through a correction of the predicted noise, without imposing unnecessary constraints on the noisy intermediate states. Finally, the reverse transition in Eq. (16) is modified to

22 $${p}_{\theta }({{{\mathcal{V}}}}_{t-1}| {{{\mathcal{V}}}}_{t})={{\mathcal{N}}}\left({{{\mathcal{V}}}}_{t-1};{\tilde{\mu }}_{t}({{{\mathcal{V}}}}_{t},{{\mathcal{P}}}({{{\mathcal{V}}}}_{0,\theta })),{\tilde{\beta }}_{t}{{\bf{I}}}\right).$$

As usual, the PCDM model is trained by minimizing the mean-squared error between the true noise injected in the forward process *ϵ* and the corrected prediction *η*_*θ*_,

23 $${{{\mathcal{L}}}}_{{{\rm{PCDM}}}}={{\mathbb{E}}}_{t,{{{\mathcal{V}}}}_{0},\epsilon }\left[\parallel \epsilon -{\eta }_{\theta }({{{\mathcal{V}}}}_{t},t){\parallel }^{2}\right],$$

where *t* is sampled uniformly from {1, …, *T*}, ${{{\mathcal{V}}}}_{0} \sim q({{{\mathcal{V}}}}_{0})$, and $\epsilon \sim {{\mathcal{N}}}({{\bf{0}}},{{\bf{I}}})$. This preserves the standard DDPM denoising objective and therefore remains equivalent to maximizing the variational lower bound on the data log-likelihood^18,40^, while explicitly enforcing the a priori physical constraints. A schematic illustration of the PCDM reverse transition, together with the pseudocode for training and sampling, is provided in Fig. 2b. After training, new turbulent velocity fields are synthesized by initializing the reverse process from Gaussian noise and iteratively applying the learned physics-constrained reverse transitions. Although we focus here on incompressibility and zero-mean momentum, the same construction can in principle, be applied to other physical constraints that admit a linear projection in state space.

We note that during the preparation of this manuscript, we became aware of a recent preprint^37^, which investigates several diffusion-based approaches to enforcing divergence-free constraints. In particular, their “div_free_denoiser” model (Table 1 therein) employs a projection-based correction within the denoising step that is similar to our approach. Our application addresses an open problem in the generative modeling of rough and anisotropic 3D turbulent flows. This represents a substantial departure from the proof-of-concept setting considered in ref. ^37^, where the target fields were obtained from the temporal evolution of decaying, smooth, and isotropic 2D turbulent flows.

### Neural network architecture

**Fig. 9:**
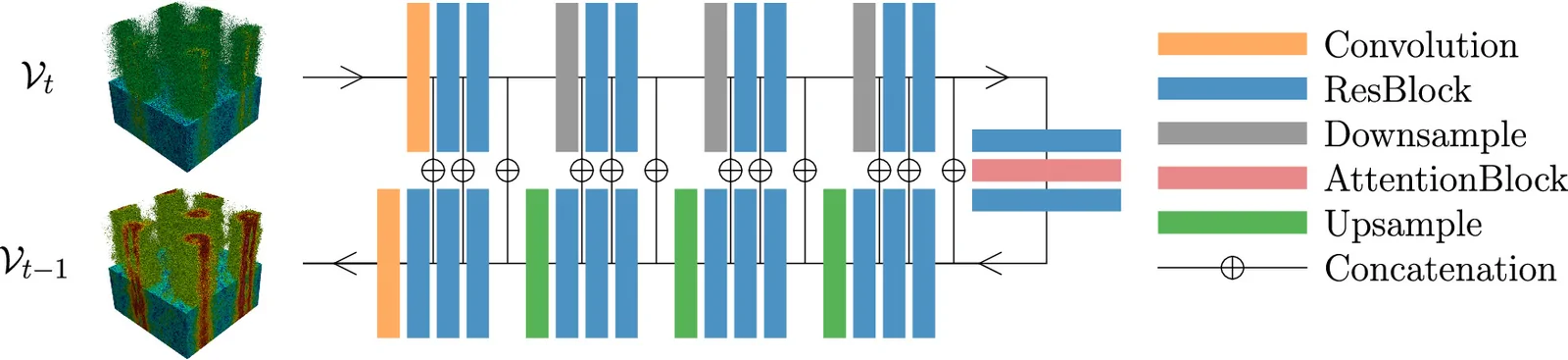
Neural network architecture. Schematic of the U-Net architecture shared in all models, including DDPM-std, DDPM-prog, and the PCDM. Different network modules are indicated in the legend.

All models reported in the main text, including DDPM-std, DDPM-prog, and the PCDM, employ the same neural network architecture. The network adopts a U-Net architecture^56^, a standard backbone in diffusion models for image synthesis^20^, adapted here to 3D velocity-field inputs, as illustrated in Fig. 9. This architecture builds upon our previous adaptation of diffusion models to 2D snapshots of rotating turbulence^27^.

The architecture consists of a contracting path and an expanding path, in which feature-map resolution is reduced and restored in the two directions perpendicular to the rotation axis, while being preserved along the rotation axis. This defines four stages of feature-map scales, at which sequences of residual blocks are applied with feature-channel widths {*C*, 2*C*, 4*C*, 8*C*} and a base width *C* = 32. The effect of varying the network capacity by changing the number of residual blocks per feature-map scale is examined in Supplementary Fig. 3. The contracting and expanding paths are connected at the bottleneck by an intermediate module consisting of two residual blocks and a four-head self-attention block. The concatenation operations in Fig. 9 denote standard U-Net skip connections, where feature tensors from the contracting path are concatenated along the channel dimension with the corresponding feature tensors in the expanding path, thereby passing high-resolution information to help recover small-scale structures. Time conditioning for the diffusion step is implemented by conditioning the network on the cumulative noise parameter ${\bar{\alpha }}_{t}$, following^57^.

The choice of preserving the feature-map resolution along the rotation axis is motivated by the quasi-two-dimensional columnar structures of rotating turbulence, which are aligned with this direction. During preliminary tests in the early stage of progressive training, we also considered a variant in which downsampling and upsampling were applied in all three spatial directions and observed comparable behavior. We used the rotation-axis-preserving version in all results reported here.

### Progressive training schedule

For the DDPM-prog baseline, we employ a progressive training schedule to alleviate the optimization difficulty associated with the extremely high dimensionality of the full 3D velocity field. In this procedure, the network architecture is kept fixed, which is possible because the model operates convolutionally and does not depend on a fixed input size, while the spatial support of the training data is gradually increased along the rotation axis.

Specifically, training is initialized on velocity fields of size 64 × 64 × *n*_*z*_, where *n*_*z*_ denotes the thickness along the rotation axis (see Fig. 4a). The value of *n*_*z*_ is progressively increased as *n*_*z*_ = 8,  16,  32,  48, and finally 64, corresponding to the full domain. At each stage, the model is trained until convergence before proceeding to the next thickness. Throughout this process, the network architecture and diffusion setup remain unchanged: increasing *n*_*z*_ changes only the input size and the corresponding feature-map size, while the trainable parameters of the convolution kernels in all blocks and of the attention layers do not depend on *n*_*z*_. As a result, the number of trainable parameters is the same at all stages.

### Diffusion setup and training configuration

All models employ the same diffusion setup with *T* = 2000 diffusion steps and a linear variance schedule, in which the noise variance increases linearly from *β*_1_ = 10^−6^ to *β*_*T*_ = 10^−2^. In the reverse transition, we follow^18^ and fix the variance to *β*_*t*_ rather than ${\tilde{\beta }}_{t}$. During training, velocity fields are augmented by random translations along the periodic directions. Optimization is performed using the AdamW optimizer with a learning rate of 10^−4^. All training and sampling are conducted on four NVIDIA A100 GPUs.

For DDPM-std and the PCDM, training is performed with a batch size of 64 for 8 × 10^5^ iterations, corresponding to approximately 12 days of training per model. For DDPM-prog, batch sizes of 256 and 128 are adopted for slab thicknesses *n*_*z*_ = 8,  16 and *n*_*z*_ = 32, respectively, while a batch size of 64 is used for *n*_*z*_ = 48 and 64. The total number of training iterations across all stages is 4 × 10^6^, corresponding to a total training time of about 56 days. For fair comparison, the checkpoints used for inference are selected after the same number of full-domain training iterations for all models, once the generated statistics have become stable, as illustrated in Fig. 4.

During inference, an exponential moving average (EMA) of the model parameters with a decay rate of 0.9999 is applied. Inference is performed with a batch size of 64. Generating 640 independent samples requires approximately 2 h.

## Supplementary information

Supplementary Information

Transparent Peer Review file

## Data Availability

The data used to train the diffusion models in this study are available for download from the open-access Smart-TURB portal at http://smart-turb.roma2.infn.it, in the TURB-Rot repository^55^. An archived version of the processed training datasets, together with the pretrained model checkpoints and generated samples used in this work, is available from the INFN OpenAccessRepository^58^. Source Data are provided with this paper.

## Code availability

The code used to train the diffusion models and to generate samples from them is publicly available at https://github.com/SmartTURB/pcdm-turb3dand archived on Zenodo^59^. A Code Ocean capsule containing the code, a test dataset, and usage instructions is available for peer review and will be made publicly available upon publication.
