# Supplementary material for: A generative diffusion framework for physically consistent 3D turbulence
Source: Nat Commun. 2026 Oct 6;17:11384. doi: 10.1038/s41467-026-78265-7 (PMC13737206; doi:10.1038/s41467-026-78265-7)
Supplement: Supplementary file 1 [file 41467_2026_78265_MOESM1_ESM.pdf]

Supplementary Information for "A Generative Diffusion Framework for Physically Consistent 3D Turbulence"

Tianyi Li,[1] Michele Buzzicotti,[1] Fabio Bonaccorso,[1] and Luca Biferale[1]

[1]*Department of Physics and INFN, University of Rome "Tor Vergata", Via della Ricerca Scientifica 1, 00133 Rome, Italy*

SUPPLEMENTARY METHODS

Forward process and reverse transition

We briefly recall the forward diffusion process of DDPM. The forward dynamics is defined as a Markov chain with Gaussian transitions

$$q(\mathcal{V}_t|\mathcal{V}_{t-1}) = \mathcal{N}\big(\mathcal{V}_t; \sqrt{1-\beta_t}\mathcal{V}_{t-1}, \beta_t\mathbf{I}\big), \tag{1}$$

where $\{\beta_t \in (0,1)\}_{t=1}^T$ is a prescribed noise schedule. Marginalizing the Markov chain yields the closed-form expression

$$q(\mathcal{V}_t|\mathcal{V}_0) = \mathcal{N}\big(\mathcal{V}_t; \sqrt{\bar{\alpha}_t}\mathcal{V}_0, (1-\bar{\alpha}_t)\mathbf{I}\big), \tag{2}$$

where $\bar{\alpha}_t = \prod_{i=1}^t (1-\beta_i)$.

During training the clean sample $\mathcal{V}_0$ is available, and the conditional posterior of the forward process can be written using Bayes' theorem as

$$q(\mathcal{V}_{t-1}|\mathcal{V}_t, \mathcal{V}_0) = \frac{q(\mathcal{V}_t|\mathcal{V}_{t-1})\, q(\mathcal{V}_{t-1}|\mathcal{V}_0)}{q(\mathcal{V}_t|\mathcal{V}_0)}. \tag{3}$$

Since all terms are Gaussian, the posterior admits the closed-form expression [1]

$$q(\mathcal{V}_{t-1}|\mathcal{V}_t, \mathcal{V}_0) = \mathcal{N}\big(\mathcal{V}_{t-1}; \tilde{\mu}_t(\mathcal{V}_t, \mathcal{V}_0), \tilde{\beta}_t\mathbf{I}\big), \tag{4}$$

with mean and variance given by

$$\tilde{\mu}_t(\mathcal{V}_t, \mathcal{V}_0) = \frac{\sqrt{\bar{\alpha}_{t-1}}\beta_t}{1-\bar{\alpha}_t}\mathcal{V}_0 + \frac{\sqrt{\alpha_t}(1-\bar{\alpha}_{t-1})}{1-\bar{\alpha}_t}\mathcal{V}_t, \tag{5}$$

$$\tilde{\beta}_t = \frac{1-\bar{\alpha}_{t-1}}{1-\bar{\alpha}_t}\beta_t. \tag{6}$$

Following [1], the reverse transition is parameterized by replacing the unknown clean sample $\mathcal{V}_0$ with its model estimate $\mathcal{V}_{0,\theta}$:

$$p_\theta(\mathcal{V}_{t-1}|\mathcal{V}_t) = \mathcal{N}\big(\mathcal{V}_{t-1}; \tilde{\mu}_t(\mathcal{V}_t, \mathcal{V}_{0,\theta}), \tilde{\beta}_t\mathbf{I}\big). \tag{7}$$

SUPPLEMENTARY DISCUSSION

Assessment of training-sample memorization

To assess whether the high fidelity of the PCDM-generated fields results from direct memorization of the training data, we compare their nearest-neighbor similarity to the training set with that of held-out DNS fields. For each query field, we identify the training snapshot with the highest cosine similarity $\mathcal{C}$, as defined in Eq. (11) of the main text. Using the 600-snapshot training set, we consider three distributions: (i) PCDM–train, obtained from 600 independently generated fields; (ii) test–train, obtained from 600 held-out DNS fields randomly selected from a later, non-overlapping segment of the same simulation; and (iii) train–train, obtained by comparing each training snapshot with the remaining 599 training snapshots in a leave-one-out manner.

As shown in Supplementary Figure 1, the PCDM–train and test–train distributions closely agree. The generated fields are therefore not more similar to the training data than unseen DNS fields, providing no evidence of direct training-sample memorization under this metric. The train–train distribution is shifted toward moderately higher similarity, possibly because snapshots from the same time segment share slowly evolving large-scale structures despite being statistically decorrelated.

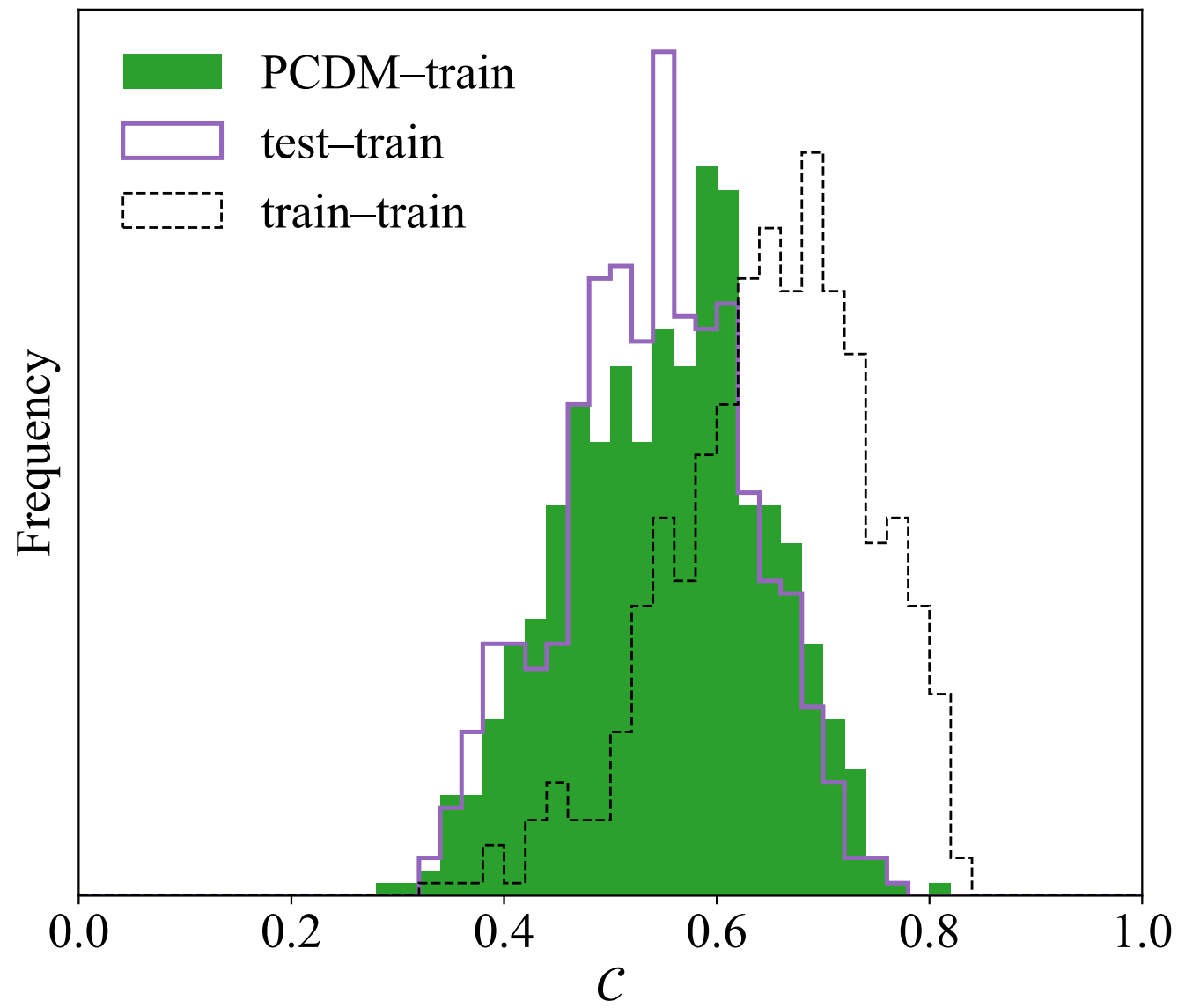

Supplementary Figure 1. **Nearest-neighbor similarity to the training set.** Histograms of the maximum cosine similarity $\mathcal{C}$ between each query velocity field and the training snapshots. The PCDM–train and test–train distributions are obtained from 600 independently generated fields and 600 held-out DNS fields, respectively. The train–train distribution is computed in a leave-one-out manner. The close agreement between the PCDM–train and test–train distributions indicates that the generated fields are not more similar to the training data than the held-out DNS fields under this metric.

Dependence on training-set size

To assess the dependence of the PCDM on the amount of training data, we train two additional models using randomly selected subsets of 152 and 300 snapshots, corresponding to approximately 25% and 50% of the full 600-snapshot training set, respectively. These models, denoted PCDM-25% and PCDM-50%, use the same network architecture and training protocol, including the same number of training iterations, as the PCDM trained on all 600 snapshots; only the training-set size is varied. Supplementary Figure 2 compares the energy spectra and scale-dependent fourth-order flatness obtained from the three models with the DNS results.

For the 2D3C component, all three models yield closely comparable energy spectra and fourth-order flatness, although agreement with the DNS spectra generally improves as the training-set size increases. The 3D fluctuating component is more sensitive to the amount of training data: reducing the training set leads to larger deviations in both the small-scale energy spectrum and the fourth-order flatness, whereas the PCDM trained on all 600 snapshots provides the most consistent agreement with the DNS. We verified that the corresponding DNS statistics remain essentially unchanged when evaluated using the reduced training subsets (not shown), indicating that the observed differences arise primarily from model training rather than from variations in the sampled training statistics. These results show that the 2D3C statistics can be learned from relatively few snapshots, while accurately reproducing the small-scale and higher-order statistics of the 3D fluctuations benefits from the full training set.

Dependence on model capacity

To assess the dependence of the PCDM on network capacity, we systematically vary the number of residual blocks while retaining the overall U-Net structure shown in Fig. 9 of the main text. The three models, denoted PCDM-NRB1, PCDM (corresponding to PCDM-NRB2), and PCDM-NRB3, contain one, two, and three residual blocks, respectively, at each feature-map scale in the contracting path, with one additional residual block at each corresponding scale in the expanding path. Here, NRB denotes the number of residual blocks per feature-map scale in the contracting path, and PCDM is the reference model used throughout the main text. The three models contain approximately 28.2, 40.4, and 52.6 million trainable parameters, respectively. They use the same training protocol, including the same number of training iterations. The corresponding training times are approximately 8.7, 11.8, and 15.2 days, respectively, on four NVIDIA A100 GPUs. Supplementary Figure 3 compares the energy spectra and scale-dependent fourth-order flatness obtained from the three models with the DNS results.

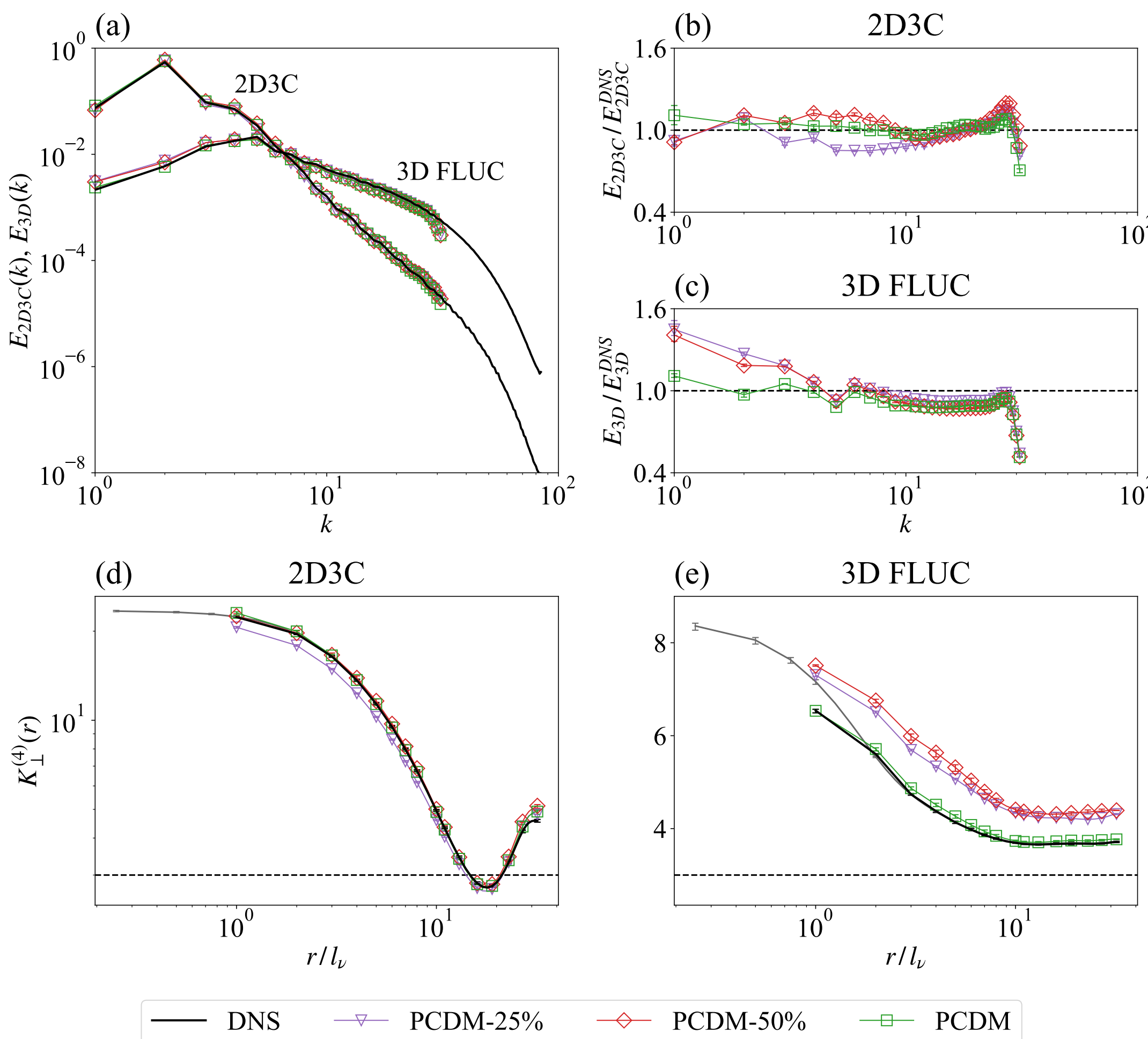

Supplementary Figure 2. **Dependence on training-set size.** Comparison of three PCDM variants trained using 25%, 50%, and 100% of the full training set, denoted PCDM-25%, PCDM-50%, and PCDM, respectively. (a) Energy spectra of the 2D3C component and the three-dimensional fluctuating field (3D FLUC), with the DNS reference shown by the black solid line. (b) Ratio of generated to DNS spectra for the 2D3C component. (c) Same as (b), but for the 3D fluctuating component. (d,e) Fourth-order flatness $K_{\perp}^{(4)}(r)$ of transverse velocity increments for (d) the 2D3C component and (e) the 3D fluctuating component, shown as a function of the separation r/l_{ν}, where l_{ν} denotes the Kolmogorov scale; the increments are measured in the plane normal to the rotation axis. In (d,e), the dashed horizontal line indicates the Gaussian reference value, and the gray curve corresponds to the full-resolution DNS data (256^3; see Methods of the main text). In all panels, error bars are of the order of the symbol size and indicate variability estimated over three independent batches, each consisting of 200 generated fields.

PCDM-NRB1 broadly reproduces the statistics of the 2D3C component but exhibits larger deviations in the energy spectrum and in the fourth-order flatness at small scales. The discrepancies become more pronounced for the weaker 3D fluctuating component, particularly in the small-scale spectrum and the scale dependence of the flatness. In contrast, PCDM and PCDM-NRB3 provide comparably accurate results for both components, although PCDM-NRB3 exhibits slightly larger deviations from the DNS reference. These results indicate that reducing the model capacity below that of the reference PCDM compromises the reproduction of multiscale statistics, while increasing the capacity beyond the reference model offers no systematic improvement in accuracy despite the additional computational cost.

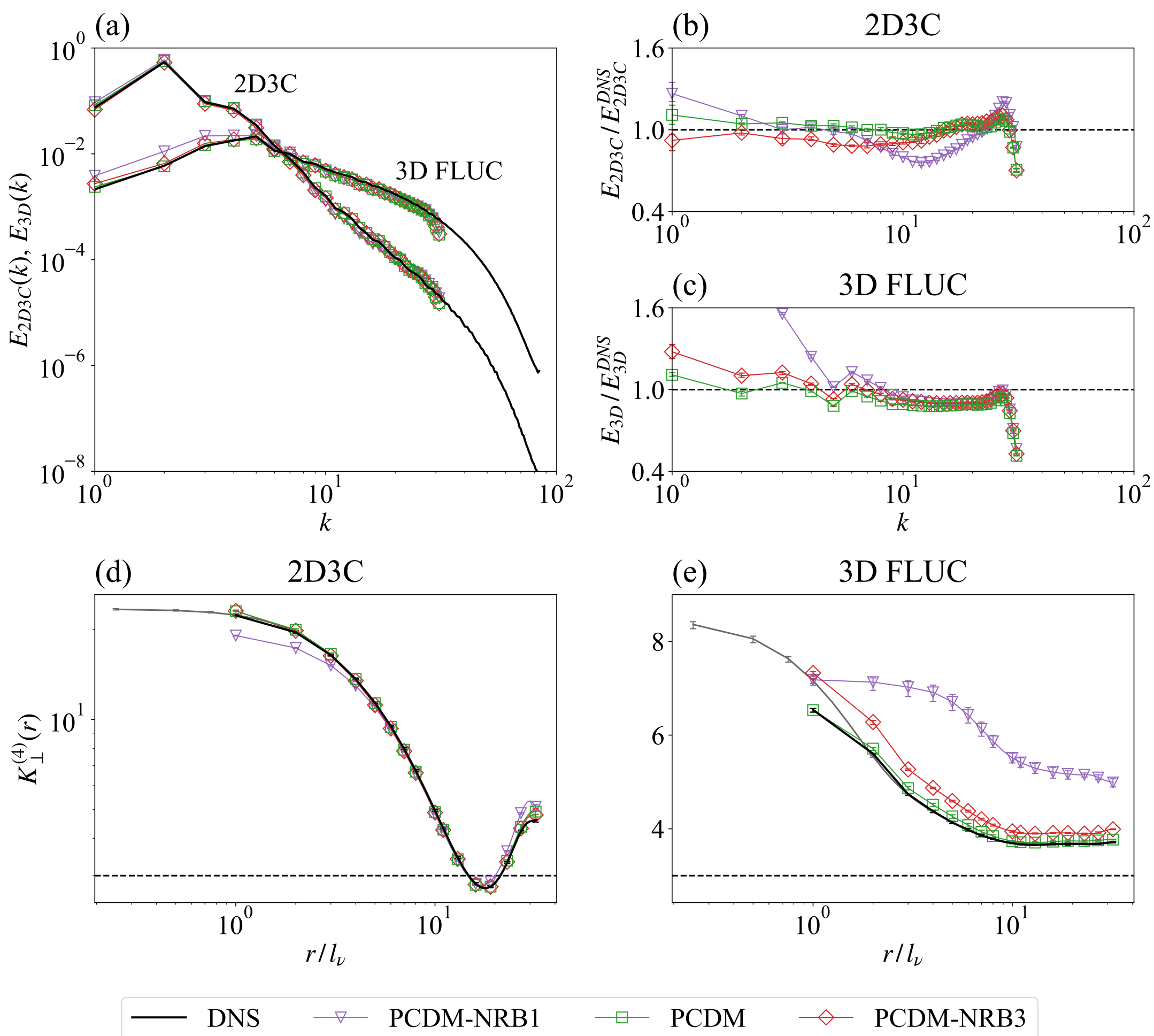

Supplementary Figure 3. **Dependence on model capacity.** Comparison of PCDM-NRB1, PCDM, and PCDM-NRB3, which contain one, two, and three residual blocks, respectively, at each feature-map scale in the contracting path, with one additional block at each corresponding scale in the expanding path. (a–e) Energy spectra, ratios of generated to DNS spectra, and fourth-order flatness of transverse velocity increments for the 2D3C and 3D fluctuating components, as defined in Supplementary Figure 2. The reference curves and error bars follow the same conventions as in Supplementary Figure 2.

[1] Ho, J., Jain, A. & Abbeel, P. Denoising diffusion probabilistic models. *Advances in neural information processing systems* **33**, 6840–6851 (2020).
