# Supplementary material for: A generative diffusion framework for physically consistent 3D turbulence
Source: Nat Commun. 2026 Oct 6;17:11384. doi: 10.1038/s41467-026-78265-7 (PMC13737206; doi:10.1038/s41467-026-78265-7)
Supplement: Supplementary file 2 [file 41467_2026_78265_MOESM2_ESM.pdf]

nature portfolio

Peer Review File

A Generative Diffusion Framework for Physically Consistent 3D Turbulence

Corresponding Author: Dr Michele Buzzicotti

This file contains all reviewer reports in order by version, followed by all author rebuttals in order by version.

Attachments originally included by the reviewers as part of their assessment can be found at the end of this file.

Version 0:

Reviewer comments:

Reviewer #1

(Remarks to the Author)
See the attachment

(Remarks on code availability)

Reviewer #2

(Remarks to the Author)
The manuscript "A Generative Diffusion Framework for Physically Consistent 3D Turbulence" by Li et al. presents a physics-constrained diffusion model (PCDM) to generate synthetic turbulent fields. The model is applied to incompressible rotating turbulence in a triple-periodic domain, a test case exhibiting physically relevant turbulence properties such as dual energy cascade (resulting from forcing at specific wave number) and intermittency. The methodological novelty lies in enforcing the incompressibility and zero-mean velocity constraints by projecting the denoised field, thus constricting the learning of the noise correction η_θ to satisfy such conditions.

The authors acknowledge that the recent work by Genuist et al. (ref. 33) from 27th January 2026 (note that the present manuscript dates from 13th March 2026) also proposes a similar method, but applied to a simpler 2D flow field. Therefore, in my honest opinion, the authors still merit credit for the proposed idea.

In general, the manuscript is well written, contains clear figures, and offers a detailed analysis of the results. Also, a data/code repository is available, and the code will be publicly shared upon publication, which is very much appreciated. Still, I would like the authors to address the following points before I can recommend the manuscript for publication in Nature Communications:

1. The literature review seems to omit relevant work in the field of synthetic turbulence generation such as the one from J. Jimenez 2024 (https://doi.org/10.1017/jfm.2024.516). This work, as well as other articles cited in the present manuscript, also account for the temporal dynamics of turbulence generation. While the present model focuses on the generation of independent turbulent field samples, adding temporal dynamics makes the problem even harder, which is omitted in the current manuscript. Additionally, the full bibliography contains 26% of self-citations, which seems excessive for such a wide research topic. I invite the authors to revisit the introduction and literature review in this sense.

2. The computational cost of the model is "hidden" in Appendix D.3. It is slightly discouraging that the model requires 12 days of training + 2h of inference to generate 640 independent samples. The first question is whether the inference cost is for 64^3 or 256^3 fields. Also, while the PCDM idea is valuable and the test case is certainly non-trivial, these results demonstrate that current diffusion models are not competitive with a numerical Navier-Stokes solver (a 256^3 DOFs case can be simulated in ~O(1h) on a GPU). The authors claim that providing a method for statistical sampling of rare and extreme events is important (p. 10), so the truth is that users would still use a numerical solver instead of a diffusion model for this purpose. Therefore, I would like the authors to expand on the numerical cost comparison within the main body of the

manuscript, ideally showing the cost to generate the 600 snapshots of the dataset compared to the PCDM cost of generating a similar number of samples. This can also help to shed light into the current conclusions, where this is indeed mentioned, but no numbers are provided.

3. Similarly, how would the computational cost of the diffusion model scale when temporal dynamics are also taken into account? And how would temporal dynamics be incorporated in the current model from architectural standpoint?

4. The manuscript currently lacks the models' sensitivity wrt. neural network size (ie. number of trainable parameters), dataset size, and other hyper-parameters typically assessed on machine-learning applications. I would like the authors to expand Appendix D.1 and provide more information that justifies the decisions related to model architecture and dataset size.

5. Fig. 7 shows the divergence-free-constraint error for the different models as computed using finite differences. In p. 7, authors claim that the DDPM-prog model does not fulfil this constraint, yet the figure shows a similar trend to the DNS result with only a slightly wider tail at the discretization accuracy order. This also hints that the progressive learning is incorporating the incompressibility constraint, differently from the standard model, so I would like the authors to clarify this result.

6. The caption of Fig. 8 states that the two realizations of the integral vs Fourier PCDMs are "indistinguishable", which I think is not correct. This is also evident from the ~0.75 mean cosine similarity of the 3D velocity component in panel (c).

(Remarks on code availability)

Reviewer #3

(Remarks to the Author)
I co-reviewed this manuscript with one of the reviewers who provided the listed reports. This is part of the Nature Communications initiative to facilitate training in peer review and to provide appropriate recognition for Early Career Researchers who co-review manuscripts.

(Remarks on code availability)

Version 1:

Reviewer comments:

Reviewer #1

(Remarks to the Author)
I can recommend the manuscript for publication.

(Remarks on code availability)

Reviewer #2

(Remarks to the Author)
The authors have done a thorough revision of the manuscript and addressed most of my previous comments. My only remaining concern is about point 4, regarding the analysis of model capacity, data dependency and choice of hyperparameters.

The authors have now justified their architecture and hyperparameters selection based on their previous 2D framework of Li et al. Atmosphere 15, 60 (2024). The original manuscript did not point to this article as the authors' baseline 2D framework, now extended to 3D, and it is important. Extending such models from 2D to 3D typically can involve additional layers of complexity, especially regarding the computational cost of the method and its dependence on data.

Since one of the main outcomes of the present work is to **rapidly** generate samples of 3D turbulent flows, the mentioned "phase diagram" between data dependency, model capacity, and physical output should be, to some extent, quantified in the present manuscript. While it might be unfeasible to train larger models given the high computational cost of training (now well detailed), smaller models could be trained to infer the practical lower bound of training capacity able to produce physical outputs. A dataset-size ablation would also be desirable, again using fewer ablation points and/or training iterations to reduce cost if necessary. And last, a per-sample similarity metric between the produced snapshots and the training set of snapshots could also be considered. If the held-out DNS snapshots and the model's generated snapshots share the same similarity wrt. the training snapshots, then the model has indeed prevented overfitting. Note that this is not shown in the present metrics (eg. energy spectra or flatness are aggregated statistics). I believe these tests, which could live in the SI and summarized in the manuscript body, would help to support the validity of the method as flows with a wider range of scales are considered (thus supporting generalization).

(Remarks on code availability)
Data and code availability are well described in their respective manuscript sections.

Reviewer #3

(Remarks to the Author)
I co-reviewed this manuscript with one of the reviewers who provided the listed reports. This is part of the Nature Communications initiative to facilitate training in peer review and to provide appropriate recognition for Early Career Researchers who co-review manuscripts.

(Remarks on code availability)

Version 2:

Reviewer comments:

Reviewer #2

(Remarks to the Author)
I genuinely appreciate the new work conducted by the authors, which thoroughly assesses the method in terms of data dependency, model capacity, and overfitting. I am now confident in recommending the manuscript for publication.

(Remarks on code availability)

Open Access This Peer Review File is licensed under a Creative Commons Attribution 4.0 International License, which permits use, sharing, adaptation, distribution and reproduction in any medium or format, as long as you give appropriate credit to the original author(s) and the source, provide a link to the Creative Commons license, and indicate if changes were made.
In cases where reviewers are anonymous, credit should be given to 'Anonymous Referee' and the source.
The images or other third party material in this Peer Review File are included in the article's Creative Commons license, unless indicated otherwise in a credit line to the material. If material is not included in the article's Creative Commons license and your intended use is not permitted by statutory regulation or exceeds the permitted use, you will need to obtain permission directly from the copyright holder.
To view a copy of this license, visit https://creativecommons.org/licenses/by/4.0/

Response to Reviewer 1

Reviewer: *The authors propose a physics-constrained diffusion model (PCDM) for synthesizing fully developed three-dimensional turbulent velocity fields, in which some a priori constraints are incorporated directly into the generative dynamics. They show that the proposed framework enables stable and statistically faithful synthesis of inertial-range three-dimensional turbulent velocity fields, accurately reproducing anisotropic energy spectra, intermittent statistics, and physical constraints in rotating turbulence. They also observe that standard de-noising diffusion probabilistic models without such constraints exhibit multiscale statistical deviations, violations of physical consistency, and substantially slower training convergence. Their results point to broader implications for generative modeling of high-dimensional, physically constrained complex systems. The paper could be considered for publication in Nature Communications after some minor revisions, which should address several issues outlined below.*

Reply: We thank the Reviewer for the positive evaluation of our work and for the constructive comments. We have carefully revised the manuscript to clarify the presentation, strengthen the discussion, and address the technical questions raised by the Reviewer. Detailed responses to each point are provided below.

Reviewer: *1. Please appropriately elaborate on the implementation process of synthesizing fully developed turbulence. It would be better to briefly discuss a comparison between the proposed method and traditional methods.*

Reply: We thank the Reviewer for these suggestions. We have revised the Methods section to make the implementation process of synthesizing fully developed turbulence more explicit and to more clearly distinguish the standard DDPM formulation from the proposed physics-constrained formulation.

We have clarified the objective at the beginning of the Methods subsection "Denoising diffusion probabilistic model":

"In the present setting, we aim to learn the distribution of instantaneous turbulent flow states, $q(\mathcal V_0)$, from DNS snapshots and to sample new synthetic velocity fields from the learned distribution."

In addition, after introducing the PCDM reverse transition and training objective, we now explicitly state how new samples are synthesized:

"After training, new turbulent velocity fields are synthesized by initializing the reverse process from Gaussian noise and iteratively applying the learned physics-constrained reverse transitions."

Regarding the comparison with traditional methods, we have revised the Introduction to better place the present work in the broader context of synthetic turbulence generation. In particular, we now cite additional representative works on reduced-order and stochastic modeling, and data-driven approaches to 3D field synthesis or spatio-temporal turbulence generation. We also clarify that the challenge addressed here is the generation of realistic instantaneous 3D turbulent velocity fields without directly solving the governing equations. The revised text reads:

"Related reduced-order, stochastic, and phenomenological modeling approaches have also been developed to describe selected statistical features and, in some cases, temporal properties of turbulent systems [13-16].

These developments raise the broader question of whether the apparent success of sophisticated models reflects the learning of genuine physical mechanisms or the reproduction of statistical properties that may already emerge from simplified stochastic descriptions. Despite decades of progress, generating realistic 3D turbulent velocity fields without directly solving the governing equations remains a challenging task due to the enormous number of active degrees of freedom distributed across a wide range of scales, strong non-Gaussian intermittency, and strict physical constraints such as incompressibility."

"A few exploratory attempts have moved toward fully 3D field synthesis or spatio-temporal turbulence generation [28-31]. The latter setting requires preserving temporal correlations and dynamically consistent evolution beyond instantaneous field statistics. Here, we focus on instantaneous 3D turbulent velocity fields, whose statistically faithful and physically consistent synthesis remains a challenging open problem."

This clarification distinguishes the present objective from DNS, which generates dynamically consistent turbulent trajectories by solving the governing equations, and from traditional synthetic approaches, which often rely on prescribed stochastic or multiscale constructions, reduced representations, or other surrogate descriptions to reproduce selected statistical features. In contrast, the present PCDM learns the single-time distribution of 3D turbulent velocity fields from DNS snapshots and is quantitatively validated against DNS statistics and physical constraints. In the Results section, we further compare the proposed PCDM with the closest data-driven baselines, DDPM-std and DDPM-prog, thereby isolating the effect of the physics-constrained formulation.

Reviewer: *2. The authors conduct an analysis of rotating turbulence and developed targeted schemes considering the effects of rotation. Please briefly discuss which innovative aspects can be extended to more general turbulence problems. What challenges will physical constraint methods face when extended to more complex turbulence problems?*

Reply: We thank the Reviewer for this question. The main transferable aspect of the present work is not specific to rotating turbulence, but rather to the incorporation of physical constraints into generative diffusion models, especially for high-dimensional turbulent systems where learning the unconstrained distribution can be fragile. In the present implementation, this is achieved by enforcing incompressibility and zero-mean momentum through an explicit projection operator. As stated in the Methods section, the same construction can in principle be applied to other physical constraints that admit a linear projection in state space.

Some architectural choices used in this work, such as preserving the feature-map resolution along the rotation axis, are specific to the present rotating-turbulence benchmark and are discussed separately in our response to Comment 9. By contrast, the physics-constrained formulation itself is more general. Potential extensions include other incompressible flow configurations, such as wall-bounded turbulence with boundary-condition and flux constraints, as well as incompressible magnetohydrodynamics, where the velocity and magnetic fields must both remain divergence-free.

At the same time, extending physical-constraint methods to more complex turbulence problems raises important challenges. The present formulation is most natural for constraints that act at the level of individual realizations and admit an explicit projection. Constraints that are nonlinear, statistical, or approximate, as well as constraints on temporal consistency across successive flow states, are more difficult to incorporate in this way.

We have clarified in the Methods subsection "Physics-constrained diffusion model" that:

“Although we focus here on incompressibility and zero-mean momentum, the same construction can in principle be applied to other physical constraints that admit a linear projection in state space.”

We have also added the following sentences to the Discussion:

“In this context, the present PCDM framework naturally invites extensions to other incompressible flow configurations, including wall-bounded turbulence with boundary-condition and flux constraints, as well as incompressible magnetohydrodynamics, where the velocity and magnetic fields must both remain divergence-free. In principle, temporal information could be incorporated by extending the generation target from a single velocity field to a short sequence of velocity fields and adopting a network architecture capable of modeling temporal correlations. This would naturally increase the computational cost, since multiple three-dimensional fields would need to be processed. However, the current formulation is tailored to constraints that act at the level of individual realizations and admit an explicit projection onto the admissible space. Extending this approach to nonlinear, statistical, or approximate constraints, as well as to temporal consistency across successive flow states, remains an important challenge for future work.”

Reviewer: *3. Equation 10 on page 4: is this equation taken in the frequency domain, or does the projection involve both Fourier transform and inverse Fourier transform?*

Reply: We thank the Reviewer for the clarification request. The projection involves both a Fourier transform and an inverse Fourier transform. To clarify this point, we have added the following sentence to the revised manuscript:

“The velocity field is first transformed to Fourier space, where the projection defined in Eq. (6) is applied, followed by an inverse Fourier transform back to physical space.”

Reviewer: *4. Equation 11 on page 4: does the resulting corrected field satisfy the two imposed physical constraints?*

Reply: We thank the Reviewer for the comment. Yes, the projected posterior estimate satisfies both imposed physical constraints. The relevant part now reads:

“Note that in Eq. (20) neither the noisy realization $\mathcal{V}_t$ nor the noise η_θ are required to satisfy the physical constraints, whereas the posterior estimate of the clean velocity field $\mathcal{P}(\mathcal{V}_{0,\theta})$ satisfies both incompressibility and zero-mean momentum.”

Reviewer: *5. Equation 12 on page 4: can an equality sign still be valid between the two sides of equation 6 after the substitution of equation 12?*

Reply: We thank the Reviewer for pointing this out. Here, Eq. (16) defines the modified reverse transition after replacing the posterior estimate with the projected counterpart. To avoid ambiguity, we have revised the wording from “becomes” to “is modified to” in the manuscript.

Reviewer: *6. In all figures, the results shown are averaged over how many flow fields, compared against how many DNS flow fields?*

Reply: We thank the Reviewer for the question. For clarity, we have added the following sentence at the beginning of the Results section:

"Unless otherwise specified, all statistical quantities reported in the following figures are computed using ensembles of 600 generated flow fields and 600 DNS reference fields, both at the Galerkin-truncated \(64^3\) resolution."

Reviewer: *7. Figure 4 on page 8: are these epochs (A,B,C) selected randomly or based on a specific criterion? Given that the loss curve exhibits significant oscillation, how to select a proper epoch for inference?*

Reply: We thank the Reviewer for the question. The labels A, B, and C correspond to training iterations (rather than epochs), and were not selected randomly or based on a specific selection criterion. Specifically, they correspond to three representative training iterations (100k, 200k, and 800k) chosen to illustrate the convergence behavior during training. To clarify this point, we have revised the figure caption accordingly:

"Three representative iterations (100k, 200k, and 800k), labeled A, B, and C, are selected from panel (b) to assess convergence."

The loss curve corresponds to the batch-averaged training loss and therefore naturally exhibits stochastic mini-batch fluctuations. In practice, inference during the late stages of training is stable, with stability further enhanced by the use of an exponential moving average (EMA) of the model parameters with a decay rate of 0.9999. Additional details are provided in the Results subsection "Spectral accuracy and training convergence" and in the Methods subsection "Diffusion setup and training configuration".

Reviewer: *8. Figure 8 on page 10: the caption states "The two realizations are visually indistinguishable". However, there are actually quite a number of noticeable differences between them. Please rephrase this sentence.*

Reply: We thank the Reviewer for the careful observation. We agree that "visually indistinguishable" was too strong. We have rephrased the statement to clarify that the two realizations show similar large-scale, 2D3C-dominated structures, while visible differences remain; in particular, the 3D fluctuating component is strongly correlated but not identical.

We have revised the corresponding statement in the Results section and in the caption as follows:

"The realizations display very similar large-scale structures, dominated by the 2D3C component, although small-scale differences are visible."

"(b) Corresponding field generated using the relaxed integral constraint formulation (PCDM-Integral). The two realizations display similar large-scale, 2D3C-dominated structures, while visible differences remain."

Reviewer: *9. Appendix D1: authors mention that the flow field is not compressed along the rotational axis dimension. Appendix D2: please give some explanations about how to gradually increase the size of this dimension without changing the model parameters?*

Reply: We thank the Reviewer for raising these implementation details. Regarding the absence of compression along the rotation axis, we have clarified in the Methods subsection "Neural network architecture" that this choice is motivated by the quasi-two-dimensional columnar structures of rotating turbulence, which are aligned with this direction.

Regarding the progressive increase of the spatial extent along the rotation axis, parameterized by \(n_z\), we have expanded the Methods subsection "Progressive training schedule" to make explicit why the model parameters remain unchanged. Since the model is convolutional and does not require a fixed input size, increasing \(n_z\) changes only the input size and the corresponding feature-map size. The trainable parameters of the convolution kernels in all blocks and of the attention layers do not depend on \(n_z\). As a result, the number of trainable parameters is the same at all stages of the progressive schedule.

We have added the following clarifications to the respective Methods subsections:

"The choice of preserving the feature-map resolution along the rotation axis is motivated by the quasi-two-dimensional columnar structures of rotating turbulence, which are aligned with this direction."

"Throughout this process, the network architecture and diffusion setup remain unchanged: increasing \(n_z\) changes only the input size and the corresponding feature-map size, while the trainable parameters of the convolution kernels in all blocks and of the attention layers do not depend on \(n_z\). As a result, the number of trainable parameters is the same at all stages."

Reviewer: *10. Figure 9 on page 13: the model involves numerous concatenation operations. please give some explanations: which tensors are concatenated, in what manner, and what is their purpose?*

Reply: We thank the Reviewer for pointing this out. The concatenation operations in Fig. 9 correspond to standard U-Net skip connections (see Ref. 56, now cited in the Methods subsection "Neural network architecture"), where feature tensors from the contracting path are concatenated along the channel dimension with the corresponding feature tensors in the expanding path. The purpose of these skip connections is to pass high-resolution information to the expanding path and help recover small-scale structures.

We have added the following clarification to the same Methods subsection:

"The concatenation operations in Fig. 9 denote standard U-Net skip connections, where feature tensors from the contracting path are concatenated along the channel dimension with the corresponding feature tensors in the expanding path, thereby passing high-resolution information to help recover small-scale structures."

Reviewer: *11. Appendix D3: authors mention that the model is trained for 800,000 epochs with a batch size of 64. please give some explanations why are so many epochs required?.*

Reply: We thank the Reviewer for the question. We first clarify that 800,000 refers to training iterations, i.e., optimizer updates, rather than epochs over the training dataset. The relatively large number of iterations is due to the nature of diffusion training: at each update, the model sees a mini-batch of velocity fields, each paired with a randomly sampled diffusion time step $t \in \{1, \ldots, T\}$, while the denoising task has to be learned over the full Markov chain with $T = 2000$ diffusion steps. Therefore, the effective training space is much larger than the number of clean velocity snapshots alone. In practice, convergence is assessed by monitoring

the stability of generated statistics, as illustrated by checkpoints A, B, and C in Fig. 4, rather than by the noisy mini-batch loss alone.

We have added the following clarification to the Methods subsection "Diffusion setup and training configuration":

"For fair comparison, the checkpoints used for inference are selected after the same number of full-domain training iterations for all models, once the generated statistics have become stable, as illustrated in Fig. 4."

Reviewer: *12. During the inference phase, when generating 640 independent samples, what is the batch size?*

Reply: We thank the Reviewer for pointing out this omission. We have clarified this point in the Methods subsection "Diffusion setup and training configuration": "Inference is performed with a batch size of 64."

Reviewer: *13. Authors propose a method for improving diffusion models based on physical constraints. Can higher-order schemes be used to achieve a better performance?*

Reply: We thank the Reviewer for the question. The proposed framework is not restricted to a specific numerical implementation of the projection operator enforcing the physical constraints. In the present work, we adopt a Fourier-space projection, which enforces incompressibility and zero-mean momentum up to machine precision, and a higher-order discretization is therefore not required for this purpose. In the Results subsection "Robustness across constraint implementations", we further show that distinct implementations of the physical constraints lead to similar generative behavior.

We have clarified this point in the revised manuscript as follows:

"The first, denoted *PCDM-Fourier*, is the formulation used throughout the preceding results and enforces incompressibility and zero-mean flow exactly through the Fourier-space projection operator defined in Eq. (6)."

Response to Reviewer 2

Reviewer: *The manuscript "A Generative Diffusion Framework for Physically Consistent 3D Turbulence" by Li et al. presents a physics-constrained diffusion model (PCDM) to generate synthetic turbulent fields. The model is applied to incompressible rotating turbulence in a triple-periodic domain, a test case exhibiting physically relevant turbulence properties such as dual energy cascade (resulting from forcing at specific wave number) and intermittency. The methodological novelty lies in enforcing the incompressibility and zero-mean velocity constraints by projecting the denoised field, thus constricting the learning of the noise correction η_θ to satisfy such conditions.*

The authors acknowledge that the recent work by Genuist et al. (ref. 33) from 27th January 2026 (note that the present manuscript dates from 13th March 2026) also proposes a similar method, but applied to a simpler 2D flow field. Therefore, in my honest opinion, the authors still merit credit for the proposed idea.

In general, the manuscript is well written, contains clear figures, and offers a detailed analysis of the results. Also, a data/code repository is available, and the code will be publicly shared upon publication, which is very much appreciated. Still, I would like the authors to address the following points before I can recommend the manuscript for publication in Nature Communications:

Reply: We thank the Reviewer for the positive evaluation of our manuscript and for the constructive comments. We are grateful that the Reviewer acknowledged the value of the proposed physics-constrained diffusion framework, and appreciated the clarity of the presentation and analysis. We have carefully revised the manuscript to expand the literature discussion, clarify the computational cost and scope of the method, and address the technical questions and clarifications raised by the Reviewer. Detailed responses to each point are provided below.

Reviewer: *1. The literature review seems to omit relevant work in the field of synthetic turbulence generation such as the one from J. Jimenez 2024 (https://doi.org/10.1017/jfm.2024.516). This work, as well as other articles cited in the present manuscript, also account for the temporal dynamics of turbulence generation. While the present model focuses on the generation of independent turbulent field samples, adding temporal dynamics makes the problem even harder, which is omitted in the current manuscript. Additionally, the full bibliography contains 26% of self-citations, which seems excessive for such a wide research topic. I invite the authors to revisit the introduction and literature review in this sense.*

Reply: We thank the Reviewer for this important comment. We agree that the previous version of the manuscript did not sufficiently acknowledge the broader literature on turbulence generation, including reduced-order or stochastic approaches and recent data-driven models that account for temporal dynamics. We have therefore revised the Introduction to clarify the relation between the present contribution and these related directions.

Specifically, we now cite reduced-order, stochastic, and phenomenological modeling approaches that aim to describe selected statistical and temporal features of turbulent systems, including the work by Jiménez and a recent spatio-temporal random-field model. We have added the following sentences in the Introduction:

"Related reduced-order, stochastic, and phenomenological modeling approaches have also been developed to describe selected statistical features and, in some cases, temporal properties of turbulent systems [13-16].

These developments raise the broader question of whether the apparent success of sophisticated models reflects the learning of genuine physical mechanisms or the reproduction of statistical properties that may already emerge from simplified stochastic descriptions."

We have also expanded the discussion of existing data-driven approaches to spatio-temporal turbulence generation. In particular, we now explicitly acknowledge recent work in this direction and clarify that incorporating temporal dynamics is substantially more challenging than the single-time setting considered here. The revised text reads:

"A few exploratory attempts have moved toward fully 3D field synthesis or spatio-temporal turbulence generation [28-31]. The latter setting requires preserving temporal correlations and dynamically consistent evolution beyond instantaneous field statistics. Here, we focus on instantaneous 3D turbulent velocity fields, whose statistically faithful and physically consistent synthesis remains a challenging open problem."

Finally, following the Reviewer's suggestion, we have revisited the bibliography to provide a broader and more balanced account of the relevant literature. We have also retained only those self-citations that are essential to the context of the present contribution.

Reviewer: *2. The computational cost of the model is "hidden" in Appendix D.3. It is slightly discouraging that the model requires 12 days of training + 2h of inference to generate 640 independent samples. The first question is whether the inference cost is for 64^3 or 256^3 fields. Also, while the PCDM idea is valuable and the test case is certainly non-trivial, these results demonstrate that current diffusion models are not competitive with a numerical Navier-Stokes solver (a 256^3 DOFs case can be simulated in ~O(1h) on a GPU). The authors claim that providing a method for statistical sampling of rare and extreme events is important (p. 10), so the truth is that users would still use a numerical solver instead of a diffusion model for this purpose. Therefore, I would like the authors to expand on the numerical cost comparison within the main body of the manuscript, ideally showing the cost to generate the 600 snapshots of the dataset compared to the PCDM cost of generating a similar number of samples. This can also help to shed light into the current conclusions, where this is indeed mentioned, but no numbers are provided.*

Reply: We thank the Reviewer for raising this issue. We have clarified that the reported inference cost refers to the Galerkin-truncated 64^3 fields used for training and statistical evaluation, rather than to the original 256^3 DNS fields. We have also added the main training and inference costs to the Results section, while keeping the full computational details in the Methods subsection "Diffusion setup and training configuration".

We would like to clarify that the present model and DNS serve different purposes. A direct wall-clock comparison is therefore not one-to-one: DNS generates temporally correlated, dynamically consistent trajectories and resolves the full range of dynamically relevant scales, whereas the trained diffusion model samples independent single-snapshot fields from the learned single-time distribution over the inertial-range scales considered here. Conversely, using DNS to obtain statistically decorrelated snapshots also requires integration over many large-eddy turnover times. In our case, the 600 decorrelated DNS snapshots span approximately 1,400 T_L, and we have added in Methods that producing these snapshots required approximately 112 wall-clock hours on 64 CPU cores, excluding the initial transient needed to reach the statistically stationary regime.

In the present setting, the value of the PCDM is to provide a physically consistent probabilistic prior for single-snapshot 3D turbulent fields. Such a prior can be used for tasks such as quality estimation and data

assimilation, for instance through guided sampling conditioned on observations. For rare and extreme events, brute-force DNS sampling can be prohibitively expensive because the relevant probabilities are very small, while obtaining rare-event statistics efficiently in complex turbulent systems remains highly non-trivial [Ragone et al., PNAS 2018]. In this context, a learned physically constrained prior could provide a useful ingredient for future rare-event exploration. We have revised the Discussion accordingly to clarify this positioning and to soften the statement on rare and extreme events.

Specifically, we made the following changes.

In the Results section, we clarified the resolution used for the generated samples:

"Unless otherwise specified, all statistical quantities reported in the following figures are computed using ensembles of 600 generated flow fields and 600 DNS reference fields, both at the Galerkin-truncated 64^3 resolution."

We also added the training and inference costs, together with the corresponding DNS cost for producing the reference dataset:

"In practice, the PCDM requires approximately 12 days of training on four NVIDIA A100 GPUs, corresponding to a roughly five-fold reduction in training time relative to DDPM-prog (see Methods for further details on the computational cost)."

"For the 64^3 fields considered here, generating 640 independent samples with any of the trained models requires approximately 2 hours on four NVIDIA A100 GPUs. For context, collecting the 600 statistically decorrelated DNS snapshots used in this work required approximately 112 wall-clock hours on 64 CPU cores (see Methods). This comparison is not one-to-one: DNS evolves temporally correlated, dynamically consistent trajectories and resolves the full range of dynamically relevant scales, whereas the trained diffusion model samples independent single-snapshot fields from the learned single-time distribution over the retained inertial-range scales."

We also report the DNS cost in the Methods subsection "Preparation of the training dataset":

"A total of 600 snapshots are collected with a temporal spacing of $\Delta t_s \approx 2.34T_L$ between successive samples to ensure statistical decorrelation, requiring approximately 112 wall-clock hours on 64 CPU cores, excluding the initial transient needed to reach the statistically stationary regime (see the inset of Fig. 1(d))."

Finally, we revised the relevant statement in the Discussion as follows:

"Our results open the way to using generative models as physically consistent probabilistic priors for single-snapshot 3D turbulent fields. Such priors may be useful for quality estimation and for data assimilation through guided sampling conditioned on observations, both of which are relevant in geophysical, environmental, and astrophysical contexts [42-47]. In addition, such priors could also support the statistical exploration of rare and extreme events in high-dimensional multiscale systems [48]."

Reviewer: *3. Similarly, how would the computational cost of the diffusion model scale when temporal dynamics are also taken into account? And how would temporal dynamics be incorporated in the current model from architectural standpoint?*

Reply: We thank the Reviewer for raising this point. In principle, temporal information could be incorporated into the present diffusion framework by extending the generated object from a single velocity field to a short sequence of velocity fields and by using a network architecture able to process temporal correlations. This would naturally increase the computational cost, since the model would need to generate and process several three-dimensional fields, rather than a single instantaneous field.

However, the main difficulty is not only architectural or computational. The present PCDM is designed for constraints that act on individual instantaneous realizations and admit an explicit projection, such as incompressibility and zero-mean momentum. By contrast, temporal dynamics impose path-level consistency conditions: successive flow states must preserve temporal correlations and be compatible with the underlying dynamical evolution. Such temporal consistency does not generally admit a simple projection analogous to the Fourier-space projection used here; enforcing it strictly would require specifying a time-discrete dynamical constraint, which depends on the temporal discretization, forcing, and numerical setup. A possible extension would therefore require combining the present instantaneous projection with additional temporal modeling across successive states, which is beyond the scope of the present single-snapshot formulation.

We have clarified this limitation and future direction in the Discussion as follows:

"In principle, temporal information could be incorporated by extending the generation target from a single velocity field to a short sequence of velocity fields and adopting a network architecture capable of modeling temporal correlations. This would naturally increase the computational cost, since multiple three-dimensional fields would need to be processed. However, the current formulation is tailored to constraints that act at the level of individual realizations and admit an explicit projection onto the admissible space. Extending this approach to nonlinear, statistical, or approximate constraints, as well as to temporal consistency across successive flow states, remains an important challenge for future work."

Reviewer: *4. The manuscript currently lacks the models' sensitivity wrt. neural network size (ie. number of trainable parameters), dataset size, and other hyper-parameters typically assessed on machine-learning applications. I would like the authors to expand Appendix D.1 and provide more information that justifies the decisions related to model architecture and dataset size.*

Reply: We thank the Reviewer for this important comment. The network architecture was chosen starting from a U-Net architecture, a standard backbone in diffusion models for image synthesis, while the diffusion setup follows standard DDPM choices. We previously adapted this framework to 2D snapshots of rotating turbulence and found that the results were robust under moderate changes of the U-Net parameters [Li et al., Atmosphere 15, 60 (2024), Appendix B]. The present model extends this design to 3D turbulent velocity fields. Because rotating turbulence contains quasi-two-dimensional columnar structures aligned with the rotation axis, we kept the feature-map resolution unchanged along this direction in the downsampling and upsampling blocks. During preliminary tests in the early stage of progressive training, we also considered a variant in which the downsampling/upsampling operations were applied in all three spatial directions, and observed comparable behavior. We used the rotation-axis-preserving version in all results reported in the manuscript.

In developing a diffusion-based generative framework for 3D turbulence, a central objective of the present work is to assess the role of explicitly incorporating physical constraints into the model. We therefore kept the architecture, diffusion setup, and training dataset fixed across all models and variants, so that the

comparison focuses on the effect of the physics-constrained formulation rather than on architecture or hyperparameter tuning.

We agree that a systematic study of the interplay between model capacity, training-set size, and the complexity of the underlying turbulent system would be highly valuable. From a physical perspective, this can be viewed as a phase diagram spanned by these three quantities, with regimes corresponding to generalization, memorization, and model failure. Such a characterization would be useful both for practical applications of generative models to turbulence and for understanding what these models learn from complex physical data. We now mention this point in the revised manuscript as an important direction for future work.

Accordingly, we have expanded the Methods subsection "Neural network architecture" as follows:

"… The network adopts a U-Net architecture [56], a standard backbone in diffusion models for image synthesis [20], adapted here to 3D velocity-field inputs, as illustrated in Fig. 9. The architecture follows our previous adaptation of diffusion models to 2D snapshots of rotating turbulence [27], where the results were found to be robust under moderate changes of the U-Net parameters.

…

The choice of preserving the feature-map resolution along the rotation axis is motivated by the quasi-two-dimensional columnar structures of rotating turbulence, which are aligned with this direction. During preliminary tests in the early stage of progressive training, we also considered a variant in which downsampling and upsampling were applied in all three spatial directions and observed comparable behavior. We used the rotation-axis-preserving version in all results reported here."

We have also added the following sentences to the Discussion section:

"More generally, a systematic characterization of the interplay between model capacity, training-set size, and the complexity of the underlying turbulent system remains an important open problem. From a physical perspective, this can be viewed as a phase diagram spanned by these quantities, with regimes corresponding to generalization, memorization, and model failure [49-52]. Such a phase diagram would be valuable for guiding practical applications of generative models to turbulence and for understanding their behavior as the complexity of the target flow increases. This issue is particularly relevant to fully developed turbulence, where the dimension of the turbulent attractor grows as $Re^{9/4}$, with Re the Reynolds number and a direct measure of the turbulent intensity [17]."

Reviewer: *5. Fig. 7 shows the divergence-free-constraint error for the different models as computed using finite differences. In p. 7, authors claim that the DDPM-prog model does not fulfil this constraint, yet the figure shows a similar trend to the DNS result with only a slightly wider tail at the discretization accuracy order. This also hints that the progressive learning is incorporating the incompressibility constraint, differently from the standard model, so I would like the authors to clarify this result.*

Reply: We thank the Reviewer for pointing this out. We agree that our previous wording should be made more precise. Fig. 7 indeed shows that DDPM-prog is much closer to DNS than DDPM-std in this finite-difference diagnostic, with only slightly broader tails. This suggests that progressive training can partially learn an approximately incompressible structure from the training data. We have revised the text to clarify that the diagnostic in Fig. 7 is computed using second-order finite differences on the Galerkin-truncated

\(64^3\) fields. This provides a fair comparison across DNS and all generative models, but it is not identical to the Fourier-space incompressibility constraint enforced exactly in the PCDM projection.

The revised text reads:

"The velocity fields generated by the PCDM are divergence-free at machine precision by construction, as a direct consequence of the physics-constrained generative dynamics. To quantify this property in a fair way, we compute a coarse-grained approximation of the gradients using second-order finite differences on the Galerkin-truncated \(64^3\) fields and compare the resulting divergence statistics among the three generative models considered here. Since this finite-difference diagnostic differs from the Fourier-space incompressibility constraint, even DNS and PCDM exhibit a finite discretization-level divergence. In Fig. 7 we show the PDF of the normalized velocity divergence,

$$\nabla \cdot \boldsymbol{u} / \langle \sum_i (\partial_i u_i)^2 \rangle^{1/2}.$$

DDPM-std exhibits substantially broader tails, indicating clear compressibility errors. DDPM-prog is much closer to the DNS reference, suggesting that progressive training can partially learn an approximately incompressible structure from the training data, although slightly broader tails remain. By contrast, PCDM agrees with the DNS up to the discretization accuracy of this diagnostic. The zero-mean momentum constraint is also not explicitly enforced in the unconstrained DDPMs, whereas both DNS and PCDM satisfy it to machine precision (not shown)."

We also clarified in the caption of Fig. 7 that the normalized velocity divergence is computed using second-order finite differences on the Galerkin-truncated \(64^3\) fields.

Reviewer: *6. The caption of Fig. 8 states that the two realizations of the integral vs Fourier PCDMs are "indistinguishable", which I think is not correct. This is also evident from the ~0.75 mean cosine similarity of the 3D velocity component in panel (c).*

Reply: We thank the Reviewer for the careful observation. We agree that "visually indistinguishable" was too strong. We have rephrased the statement to clarify that the two realizations show similar large-scale, 2D3C-dominated structures, while visible differences remain; in particular, the 3D fluctuating component is strongly correlated but not identical.

We have revised the corresponding statement in the main text and in the caption as follows:

"The realizations display very similar large-scale structures, dominated by the 2D3C component, although small-scale differences are visible."

"(b) Corresponding field generated using the relaxed integral constraint formulation (PCDM-Integral). The two realizations display similar large-scale, 2D3C-dominated structures, while visible differences remain."

Response to Reviewer 3

Reviewer: *I co-reviewed this manuscript with one of the reviewers who provided the listed reports. This is part of the Nature Communications initiative to facilitate training in peer review and to provide appropriate recognition for Early Career Researchers who co-review manuscripts.*

Reply: We thank the Reviewer for participating in the evaluation of our manuscript.

Response to Reviewer 2

Reviewer: *The authors have done a thorough revision of the manuscript and addressed most of my previous comments. My only remaining concern is about point 4, regarding the analysis of model capacity, data dependency and choice of hyperparameters.*

The authors have now justified their architecture and hyperparameters selection based on their previous 2D framework of Li et al. Atmosphere 15, 60 (2024). The original manuscript did not point to this article as the authors' baseline 2D framework, now extended to 3D, and it is important. Extending such models from 2D to 3D typically can involve additional layers of complexity, especially regarding the computational cost of the method and its dependence on data.

*Since one of the main outcomes of the present work is to **rapidly** generate samples of 3D turbulent flows, the mentioned "phase diagram" between data dependency, model capacity, and physical output should be, to some extent, quantified in the present manuscript. While it might be unfeasible to train larger models given the high computational cost of training (now well detailed), smaller models could be trained to infer the practical lower bound of training capacity able to produce physical outputs. A dataset-size ablation would also be desirable, again using fewer ablation points and/or training iterations to reduce cost if necessary. And last, a per-sample similarity metric between the produced snapshots and the training set of snapshots could also be considered. If the held-out DNS snapshots and the model's generated snapshots share the same similarity wrt. the training snapshots, then the model has indeed prevented overfitting. Note that this is not shown in the present metrics (eg. energy spectra or flatness are aggregated statistics). I believe these tests, which could live in the SI and summarized in the manuscript body, would help to support the validity of the method as flows with a wider range of scales are considered (thus supporting generalization).*

Reply: We thank the Reviewer for this constructive comment. We agree that a quantitative characterization of the dependence of the generated physical outputs on model capacity and training-set size, together with an assessment of possible training-sample memorization, is important for evaluating the validity of the proposed framework.

To address this point, we have added three complementary analyses in the Supplementary Discussion:

(1) Assessment of training-sample memorization.

We introduced a nearest-neighbor cosine-similarity analysis comparing generated fields and held-out DNS fields with respect to the training set. The similarity distribution of generated fields is comparable to that of unseen DNS fields, providing no evidence of direct training-sample memorization under this metric.

(2) Dependence on training-set size.

We trained additional PCDM models using 25% and 50% of the full training dataset while keeping the network architecture and training protocol unchanged. We found that the dominant 2D3C component remains relatively robust, whereas reproducing the small-scale and higher-order statistics of the weaker 3D fluctuating component benefits from the full training set.

(3) Dependence on model capacity.

We performed an architecture ablation by varying the number of residual blocks per feature-map scale while retaining the overall U-Net structure. Reducing the model capacity below the reference PCDM leads to larger deviations from DNS statistics, whereas increasing the capacity beyond the reference model does not provide systematic improvement despite the increased computational cost.

The main findings from these analyses have been summarized in a new Results subsection entitled "Sensitivity to training-set size and model capacity":

"To assess whether the high statistical fidelity of the PCDM-generated fields reflects learning of the underlying turbulent distribution rather than direct memorization of the training data, we perform a nearest-neighbor cosine-similarity analysis. The generated fields exhibit a similarity distribution comparable to that of held-out DNS fields, providing no evidence of direct training-sample memorization under this metric (see Supplementary Fig. 1).

We further assess the sensitivity of the generated statistics to training-set size and model capacity (Supplementary Figs. 2 and 3). Reducing the training-set size or model capacity leads to larger deviations from the DNS statistics, particularly for the weaker 3D fluctuating component, whereas the dominant 2D3C component remains comparatively robust. Increasing the capacity beyond the reference model does not yield systematic improvements despite the additional computational cost."

A reference to the model capacity analysis has been added in the "Neural network architecture" subsection of the Methods:

"The effect of varying the network capacity by changing the number of residual blocks per feature-map scale is examined in Supplementary Fig. 3."

Detailed descriptions of these analyses have been added to the Supplementary Discussion:

SUPPLEMENTARY DISCUSSION

Assessment of training-sample memorization

To assess whether the high fidelity of the PCDM-generated fields results from direct memorization of the training data, we compare their nearest-neighbor similarity to the training set with that of held-out DNS fields. For each query field, we identify the training snapshot with the highest cosine similarity $\mathcal{C}$, as defined in Eq. (11) of the main text. Using the 600-snapshot training set, we consider three distributions: (i) PCDM–train, obtained from 600 independently generated fields; (ii) test–train, obtained from 600 held-out DNS fields randomly selected from a later, non-overlapping segment of the same simulation; and (iii) train–train, obtained by comparing each training snapshot with the remaining 599 training snapshots in a leave-one-out manner.

As shown in Supplementary Fig. 1, the PCDM–train and test–train distributions closely agree. The generated fields are therefore not more similar to the training data than unseen DNS fields, providing no evidence of direct training-sample memorization under this metric. The train–train distribution is shifted toward moderately higher similarity, possibly because snapshots from the same time segment share slowly evolving large-scale structures despite being statistically decorrelated.

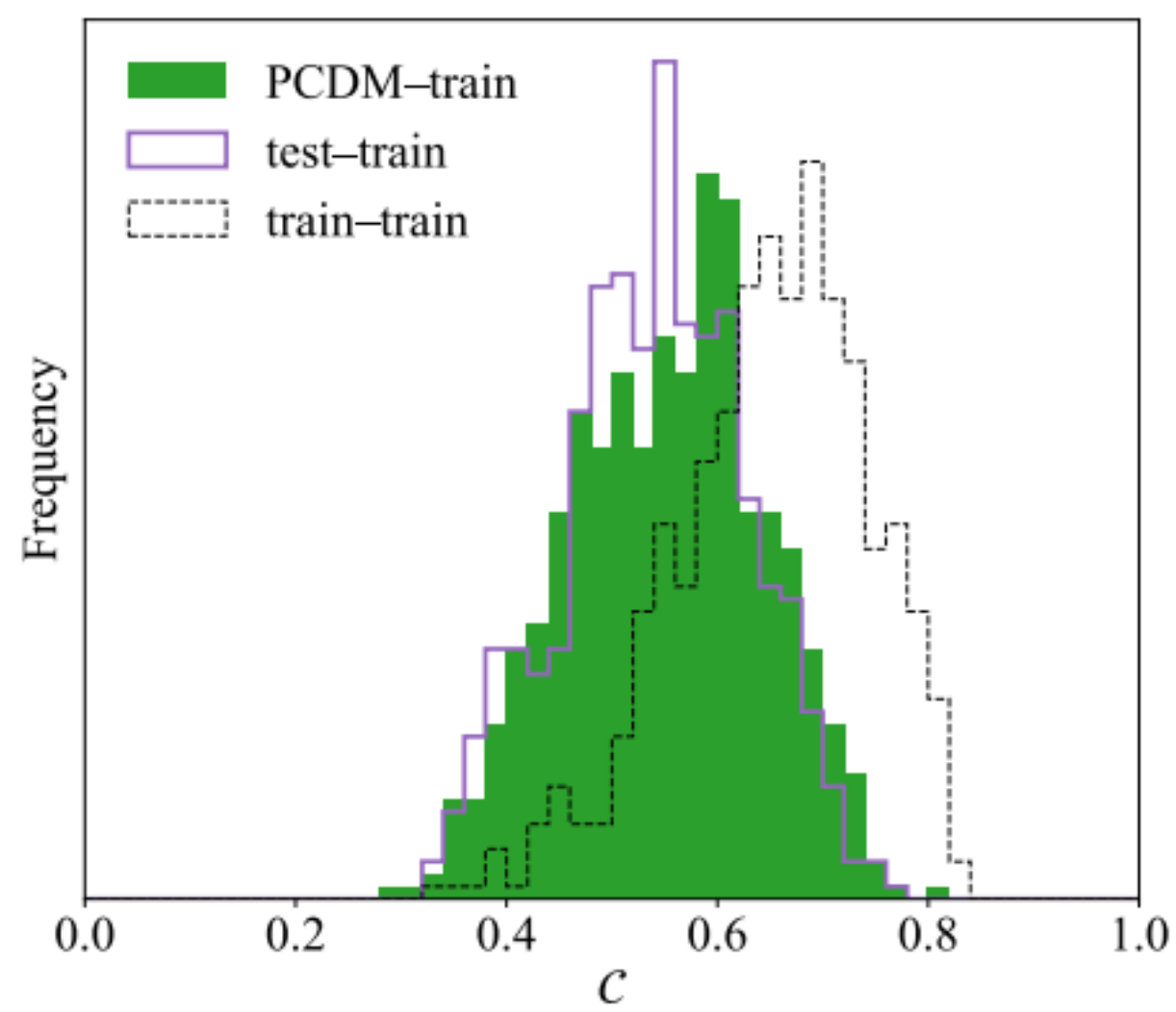

FIG. 1. **Nearest-neighbor similarity to the training set.** Histograms of the maximum cosine similarity $\mathcal{C}$ between each query velocity field and the training snapshots. The PCDM–train and test–train distributions are obtained from 600 independently generated fields and 600 held-out DNS fields, respectively. The train–train distribution is computed in a leave-one-out manner. The close agreement between the PCDM–train and test–train distributions indicates that the generated fields are not more similar to the training data than the held-out DNS fields under this metric.

Dependence on training-set size

To assess the dependence of the PCDM on the amount of training data, we train two additional models using randomly selected subsets of 152 and 300 snapshots, corresponding to approximately 25% and 50% of the full 600-snapshot training set, respectively. These models, denoted PCDM-25% and PCDM-50%, use the same network architecture and training protocol, including the same number of training iterations, as the PCDM trained on all 600 snapshots; only the training-set size is varied. Supplementary Fig. 2 compares the energy spectra and scale-dependent fourth-order flatness obtained from the three models with the DNS results.

For the 2D3C component, all three models yield closely comparable energy spectra and fourth-order flatness, although agreement with the DNS spectra generally improves as the training-set size increases. The 3D fluctuating component is more sensitive to the amount of training data: reducing the training set leads to larger deviations in both the small-scale energy spectrum and the fourth-order flatness, whereas the PCDM trained on all 600 snapshots provides the most consistent agreement with the DNS. We verified that the corresponding DNS statistics remain essentially unchanged when evaluated using the reduced training subsets (not shown), indicating that the observed differences arise primarily from model training rather than from variations in the sampled training statistics. These results show that the 2D3C statistics can be learned from relatively few snapshots, while accurately reproducing the small-scale and higher-order statistics of the 3D fluctuations benefits from the full training set.

Dependence on model capacity

To assess the dependence of the PCDM on network capacity, we systematically vary the number of residual blocks while retaining the overall U-Net structure shown in Fig. 9 of the main text. The three models, denoted PCDM-NRB1, PCDM (corresponding to PCDM-NRB2), and PCDM-NRB3, contain one, two, and three residual blocks, respectively, at each feature-map scale in the contracting path, with one additional residual block at each corresponding scale in the expanding path. Here, NRB denotes the number of residual blocks per feature-map scale in the contracting path, and PCDM is the reference model used throughout the main text. The three models contain approximately 28.2, 40.4, and 52.6 million trainable parameters, respectively. They use the same training protocol, including the same number of training iterations. The corresponding training times are approximately 8.7, 11.8, and 15.2 days, respectively, on four NVIDIA A100 GPUs. Supplementary Fig. 3 compares the energy spectra and scale-dependent fourth-order flatness obtained from the three models with the DNS results.

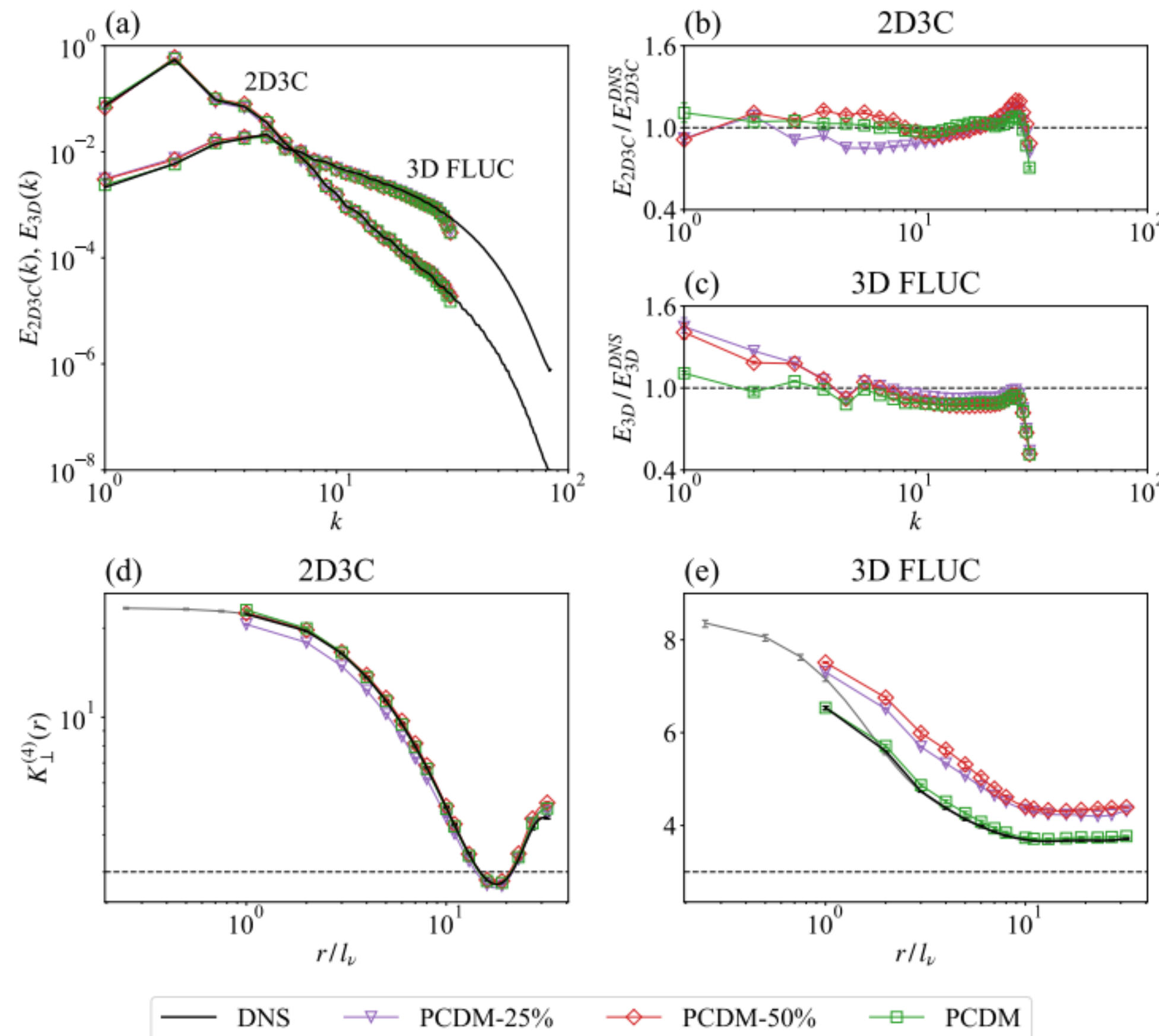

FIG. 2. **Dependence on training-set size.** Comparison of three PCDM variants trained using 25%, 50%, and 100% of the full training set, denoted PCDM-25%, PCDM-50%, and PCDM, respectively. (a) Energy spectra of the 2D3C component and the three-dimensional fluctuating field (3D FLUC), with the DNS reference shown by the black solid line. (b) Ratio of generated to DNS spectra for the 2D3C component. (c) Same as (b), but for the 3D fluctuating component. (d,e) Fourth-order flatness $K_{\perp}^{(4)}(r)$ of transverse velocity increments for (d) the 2D3C component and (e) the 3D fluctuating component, shown as a function of the separation r/l_{ν}, where l_{ν} denotes the Kolmogorov scale; the increments are measured in the plane normal to the rotation axis. In (d,e), the dashed horizontal line indicates the Gaussian reference value, and the gray curve corresponds to the full-resolution DNS data (256^3; see Methods of the main text). In all panels, error bars are of the order of the symbol size and indicate variability estimated over three independent batches, each consisting of 200 generated fields.

PCDM-NRB1 broadly reproduces the statistics of the 2D3C component but exhibits larger deviations in the energy spectrum and in the fourth-order flatness at small scales. The discrepancies become more pronounced for the weaker 3D fluctuating component, particularly in the small-scale spectrum and the scale dependence of the flatness. In contrast, PCDM and PCDM-NRB3 provide comparably accurate results for both components, although PCDM-NRB3 exhibits slightly larger deviations from the DNS reference. These results indicate that reducing the model capacity below that of the reference PCDM compromises the reproduction of multiscale statistics, while increasing the capacity beyond the reference model offers no systematic improvement in accuracy despite the additional computational cost.

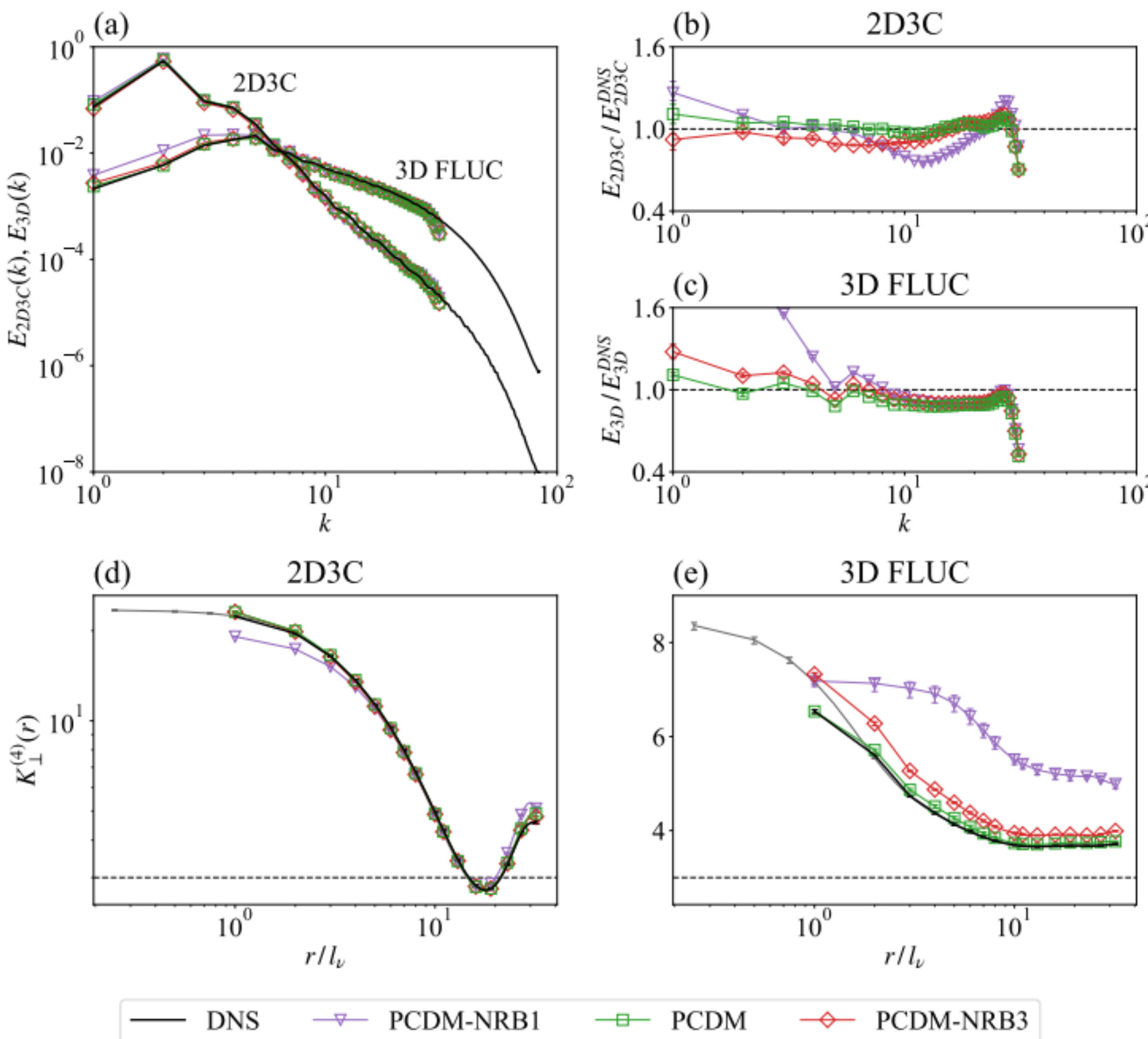

FIG. 3. **Dependence on model capacity.** Comparison of PCDM-NRB1, PCDM, and PCDM-NRB3, which contain one, two, and three residual blocks, respectively, at each feature-map scale in the contracting path, with one additional block at each corresponding scale in the expanding path. (a–e) Energy spectra, ratios of generated to DNS spectra, and fourth-order flatness of transverse velocity increments for the 2D3C and 3D fluctuating components, as defined in Supplementary Fig. 2. The reference curves and error bars follow the same conventions as in Supplementary Fig. 2.

Report to the manuscript NCOMMS-26-028253 titled “A Generative Diffusion Framework for Physically Consistent 3D Turbulence”

The authors propose a physics-constrained diffusion model (PCDM) for synthesizing fully developed three-dimensional turbulent velocity fields, in which some a priori constraints are incorporated directly into the generative dynamics. They show that the proposed framework enables stable and statistically faithful synthesis of inertial-range three-dimensional turbulent velocity fields, accurately reproducing anisotropic energy spectra, intermittent statistics, and physical constraints in rotating turbulence. They also observe that standard de-noising diffusion probabilistic models without such constraints exhibit multi-scale statistical deviations, violations of physical consistency, and substantially slower training convergence. Their results point to broader implications for generative modeling of high-dimensional, physically constrained complex systems. The paper could be considered for publication in Nature Communications after some minor revisions, which should address several issues outlined below.

1. Please appropriately elaborate on the implementation process of synthesizing fully developed turbulence. It would be better to briefly discuss a comparison between the proposed method and traditional methods.

2. The authors conduct an analysis of rotating turbulence and developed targeted schemes considering the effects of rotation. Please briefly discuss which innovative aspects can be extended to more general turbulence problems. What challenges will physical constraint methods face when extended to more complex turbulence problems?

3. Equation 10 on page 4: is this equation taken in the frequency domain, or does the projection involve both Fourier transform and inverse Fourier transform?

4. Equation 11 on page 4: does the resulting corrected field satisfy the two imposed physical constraints?

5. Equation 12 on page 4: can an equality sign still be valid between the two sides of equation 6 after the substitution of equation 12?

6. In all figures, the results shown are averaged over how many flow fields, compared against how many DNS flow fields?

7. Figure 4 on page 8: are these epochs (A,B,C) selected randomly or based on a specific criterion? Given that the loss curve exhibits significant oscillation, how to select a proper epoch for inference?

8. Figure 8 on page 10: the caption states “The two realizations are visually indistinguishable”. However, there are actually quite a number of noticeable

differences between them. Please rephrase this sentence.

9. Appendix D1: authors mention that the flow field is not compressed along the rotational axis dimension. Appendix D2: please give some explanations about how to gradually increase the size of this dimension without changing the model parameters?

10. Figure 9 on page 13: the model involves numerous concatenation operations. please give some explanations: which tensors are concatenated, in what manner, and what is their purpose?

11. Appendix D3: authors mention that the model is trained for 800,000 epochs with a batch size of 64. please give some explanations why are so many epochs required?.

12. During the inference phase, when generating 640 independent samples, what is the batch size?

13. Authors propose a method for improving diffusion models based on physical constraints. Can higher-order schemes be used to achieve a better performance?
